# Stabilization of N_6_ and N_8_ anionic units and 2D polynitrogen layers in high-pressure scandium polynitrides

**DOI:** 10.1038/s41467-024-46313-9

**Published:** 2024-03-12

**Authors:** Andrey Aslandukov, Alena Aslandukova, Dominique Laniel, Saiana Khandarkhaeva, Yuqing Yin, Fariia I. Akbar, Stella Chariton, Vitali Prakapenka, Eleanor Lawrence Bright, Carlotta Giacobbe, Jonathan Wright, Davide Comboni, Michael Hanfland, Natalia Dubrovinskaia, Leonid Dubrovinsky

**Affiliations:** 1 Bavarian Research Institute of Experimental Geochemistry and Geophysics (BGI), University of Bayreuth, 95440 Bayreuth, Germany; 2 Material Physics and Technology at Extreme Conditions, Laboratory of Crystallography, University of Bayreuth, 95440 Bayreuth, Germany; 3 Centre for Science at Extreme Conditions and School of Physics and Astronomy, University of Edinburgh, EH9 3FD Edinburgh, United Kingdom; 4 Department of Physics, Chemistry and Biology (IFM), Linköping University, SE-581 83 Linköping, Sweden; 5 Center for Advanced Radiation Sources, University of Chicago, Chicago, IL 60637 USA; 6 European Synchrotron Radiation Facility, 38000 Grenoble, France

**Keywords:** Physical chemistry, Solid-state chemistry, Materials chemistry, Solid-phase synthesis

## Abstract

Nitrogen catenation under high pressure leads to the formation of polynitrogen compounds with potentially unique properties. The exploration of the entire spectrum of poly- and oligo-nitrogen moieties is still in its earliest stages. Here, we report on four novel scandium nitrides, Sc_2_N_6_, Sc_2_N_8_, ScN_5,_ and Sc_4_N_3_, synthesized by direct reaction between yttrium and nitrogen at 78-125 GPa and 2500 K in laser-heated diamond anvil cells. High-pressure synchrotron single-crystal X-ray diffraction reveals that in the crystal structures of the nitrogen-rich Sc_2_N_6_, Sc_2_N_8,_ and ScN_5_ phases nitrogen is catenated forming previously unknown N_6_^6^^−^ and N_8_^6^^−^ units and ${\!\,}_{\infty }{\!\,}^{2}({{{{{\rm{N}}}}}}_{5}^{3-})$ anionic corrugated 2D-polynitrogen layers consisting of fused N_12_ rings. Density functional theory calculations, confirming the dynamical stability of the synthesized compounds, show that Sc_2_N_6_ and Sc_2_N_8_ possess an anion-driven metallicity, while ScN_5_ is an indirect semiconductor. Sc_2_N_6_, Sc_2_N_8_, and ScN_5_ solids are promising high-energy-density materials with calculated volumetric energy density, detonation velocity, and detonation pressure higher than those of TNT.

## Abstract (web summary)

The authors discover high-pressure Sc_2_N_6_, Sc_2_N_8_, and ScN_5_ polynitrides, synthesized at high pressure and temperature conditions, featuring a unique nitrogen catenation and previously unknown N_6_^6^^−^ anions, N_8_^6^^−^ anions and anionic corrugated 2D-polynitrogen layers.

## Introduction

The discovery of nitrogen polymerization under high pressures has significantly extended the nitrogen chemistry. While the polymeric single-bonded nitrogen allotropes are formed only at pressures above 110 GPa^1–3^, the introduction of electropositive elements facilitates breaking the N_2_ triple-bond and initiates nitrogen catenation at significantly lower pressures. Indeed, under high-pressure high-temperature conditions nitrogen easily reacts with metals and forms numerous compounds featuring charged nitrogen N_2_^*x*^^−^ dimers^4–19^ at low-to-mild pressures (5-50 GPa), or various catenated nitrogen units (*e.g*. tetranitrogen N_4_^4^^−^ units^20^, pentazolate N_5_^−^ rings^21–23^, N_6_ rings^24–26^, and N_18_ macrocycle^27^) and 1D-polynitrogen chains^20,28–33^ at mild-to-high pressures (>50 GPa). Some of the nitrogen species discovered under high-pressure (*e.g.* pentazolate-anion, whose first stabilization in bulk was achieved in CsN_5_ at 60 GPa^22^) were subsequently synthesized by conventional chemistry methods under ambient pressure^34–36^.

In addition to the discoveries of unique nitrogen entities that push the boundaries of fundamental nitrogen chemistry, nitrides and polynitrides synthesized under high pressure often possess key properties for functional applications such as high hardness^7^, unique electronic properties^33^, and high energy density^37^. Polynitrides with a high nitrogen content are especially promising as high-energy-density materials (HEDM) because their decomposition results in the formation of molecular nitrogen, which is accompanied by a large energy release. The latter is due to a large difference between the energy of the triple intramolecular bond in N_2_ and the energy of double and single bonds in polynitrogen units^37^. For HEDMs the molecular weight of the compound also matters: with other properties being similar, the lighter the elements in the solid, the higher the gravimetric energy density of the compound. Since scandium is the lightest transition metal, its polynitrides may be especially promising as HEDM.

Hitherto, only one binary Sc-N compound is known: cubic scandium nitride ScN with the rock salt structure, which exists at ambient conditions and is predicted to be stable up to ~250 GPa^38^. There are several theoretical studies^39–42^, where nitrogen-rich phases with ScN_3_, ScN_5_, ScN_6_, and ScN_7_ compositions have been predicted to be stable under 30–110 GPa and may have potential as HEDM (gravimetric energy density ranges from 2.40 kJ/g to 4.23 kJ/g).

In this study, we experimentally investigated the behavior of the Sc-N system at pressures between 50 to 125 GPa and high temperatures. Here we present the synthesis and characterization of four novel Sc-N phases, whose structures were solved and refined on the basis of single-crystal X-ray diffraction. The nitrogen-rich polynitrides Sc_2_N_6_, Sc_2_N_8_, and ScN_5_ feature a unique nitrogen catenation: previously unknown N_6_^6^^−^ and N_8_^6^^−^ nitrogen units and ${\!\,}_{\infty }{\!\,}^{2}({{{{{\rm{N}}}}}}_{5}^{3-})$ anionic 2D-polynitrogen layers consisting of fused N_12_ rings, respectively.

## Results and discussion

**Fig. 1:**
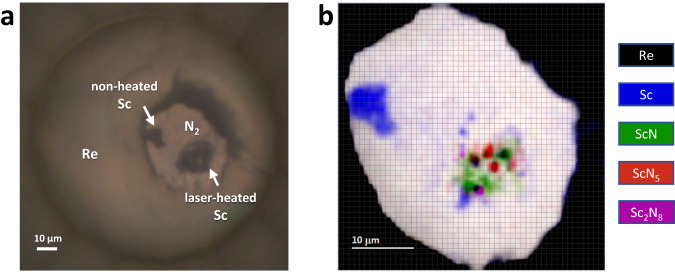
Sample chamber of the diamond anvil cell at 96 GPa. **a** Micro-photo of the sample chamber. **b** 2D X-ray diffraction map (collected with 0.75 µm steps at the ID11 beamline of the ESRF) showing the distribution of the scandium nitrides phases (determined by single-crystal XRD) within the heated sample at 96 GPa. The color intensity is proportional to the intensity of the following reflections: the (1 1 1) and (3 3 1) of ScN for the green regions; the (1 1 1) of ScN_5_ for the red regions; the (0 2 1) of Sc_2_N_8_ for the purple regions.

In this study diamond anvil cells (DACs) loaded with scandium pieces embedded in molecular nitrogen were used (see Methods section for details). Samples were compressed to their target pressures and laser-heated at 2500(300) K. Laser-heating experiments were carried out at pressures of 50(1), 78(2), 96(2), and 125(2) GPa (Supplementary Table 1). After laser-heating, detailed X-ray diffraction maps were collected around the heated spot to pinpoint the location of crystallites most appropriate for single-crystal X-ray diffraction measurements (Fig. 1). Then single-crystal X-ray diffraction data (Supplementary Fig. 1) were collected at the selected positions to identify the phases’ crystal structure and chemical composition.

According to the synchrotron single-crystal X-ray diffraction data, only the well-known ScN phase (rock-salt type structure, *a* = 4.2492(7) Å, V = 76.72(4) Å^3^ at 50 GPa) was formed at 50 GPa. The obtained volume is in good agreement with the published ScN equation of state^38^. At 78 GPa, two novel scandium nitrides with chemical formulas Sc_2_N_6_ and Sc_2_N_8_ were obtained along with ScN. At 96 GPa, a mixture of ScN, Sc_2_N_8,_ as well as the previously unobserved ScN_5_, was obtained. And, finally, at 125 GPa the collected synchrotron single-crystal X-ray diffraction data and the subsequent crystal structure solution and refinement revealed the formation of the ScN_5_ and Sc_4_N_3_ phases. Overall four novel Sc-N phases were synthesized by chemical reactions of Sc and N_2_ at 2500 K in the pressure range of 78 to 125 GPa (Supplementary Fig. 2).

Remarkably, at 50 GPa, scandium behaves like at ambient pressure producing only ScN, while at higher pressures a rich variety of phases was observed. In addition to a significant increase in the chemical potential of nitrogen under high pressure^43^, another possible reason explaining such difference in chemistry between 50 and 78 GPa is a significant drop in scandium’s electronegativity at 60 GPa (Supplementary Fig. 3a) and as a result, scandium is predicted to be the least electronegative atom in 60–110 GPa pressure range^44^. It leads to the significant increase of difference in electronegativity between N and Sc above 60 GPa (Supplementary Fig. 3b), which increases the chemical reactivity of scandium, decreases the potential kinetic barriers of reactions, and leads to the appearance of more local minima in the energy landscape.

The refinement against single-crystal X-ray diffraction data for all synthesized compounds resulted in very good agreement factors (Supplementary Tables 2–6). For cross-validation of the crystal structures, we performed density functional theory (DFT) calculations using the Vienna ab initio simulation package^45^ (see Methods section for details). We carried out variable cell structural relaxations for Sc_2_N_6_, Sc_2_N_8_, and ScN_5_ and found that the relaxed structural parameters closely reproduce the corresponding experimental values (Supplementary Tables 7–9).

**Fig. 2:**
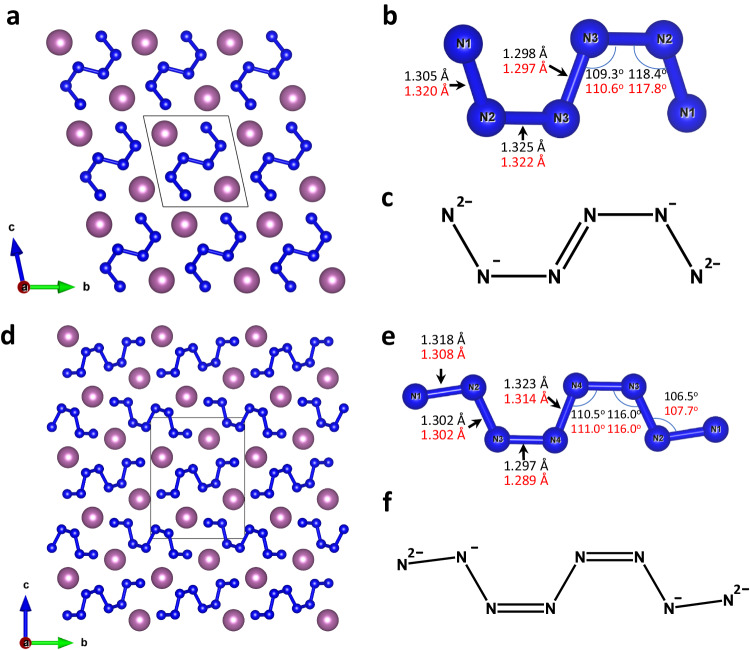
Crystal structure of Sc_2_N_6_ and Sc_2_N_8_ at 78 GPa. **a** A view of Sc_2_N_6_ along the *a*-axis; **b** an N_6_ unit; **c** structural formula of an N_6_ unit; **d** a view of Sc_2_N_8_ along the *a*-axis; **e** an N_8_ unit; **f** structural formula of an N_8_ unit. Sc atoms are purple, N atoms are blue; thin grey lines outline the unit cell. Values of bond lengths and angles obtained from the experiment are shown in black, while those obtained from the DFT calculations are shown in red.

Sc_2_N_6_ synthesized at 78 GPa (Fig. 2a) crystalizes in the triclinic crystal system (space group *P*−1 (#2)). The structure of Sc_2_N_6_ has one Sc and three N distinct atomic positions (see Supplementary Table 3 and the CIF for the full crystallographic data). Nitrogen atoms form isolated “zig-zag” N_6_ units (Fig. 2a, b). The existence of this phase was predicted at pressures of 30–100 GPa^39^.

The structure of Sc_2_N_8_ (Fig. 2d) has the monoclinic space group *P*2_1_/*c* (#14) with one Sc and four N distinct atomic positions (see Supplementary Table 4 and the CIF for the full crystallographic data). Nitrogen atoms form isolated “zig-zag” N_8_ units (Fig. 2d, e) that have never been observed or predicted.

The bond length analysis of the N_6_ and N_8_ units suggests that N1-N2, N2-N3 (in N_6_ unit) and N1-N2, N2-N3, N4-N4 (in N_8_ unit) are single-bonded, while N3-N4 (in N_6_ unit) and N3-N4 (in N_8_ unit) are double-bonded (Fig. 2b,c,e,f). Then, the charges can be described in a classic ionic approach: the total charge of [N_6_]^6^^−^ and [N_8_]^6^^−^ units is 6-, which corresponds to the +3 oxidation state of Sc atoms. The angle values and a small difference in bond length indicate the strong electron delocalization (indeed several different resonance Lewis formulas can be drawn for N_6_ and N_8_) and nitrogen atoms cannot be considered as purely *sp*^2^ or *sp*^3^ hybridized.

**Fig. 3:**
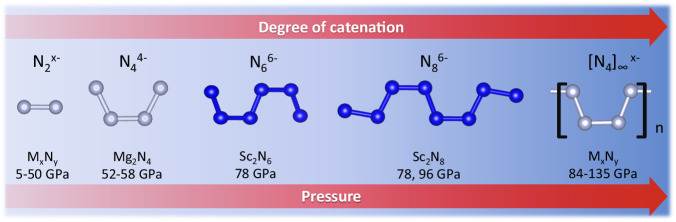
Experimentally observed catenated nitrogen units and chains. The units in blue were first discovered in the present study.

The two novel catenated nitrogen units N_6_^6^^−^ and N_8_^6^^−^ discovered in this study—being intermediate non-cyclic species between dinitride and 1D-polynitrogen anions—significantly expand the list of anionic nitrogen oligomers (Fig. 3). Notably, all these units are built of an even number of nitrogen atoms suggesting their formation via the polymerization of dinitrogen molecules. The degree of polymerization increases with pressure: dinitrides are synthesized at low pressures (<50 GPa); N_4_, N_6_, N_8_ units are obtained at mild pressures (50–80 GPa), while 1D-polynitrogen chains are usually formed above 100 GPa. Since the dinitrogen ([N_2_]^*x*^^−^ x = 0.66, 0.75, 1, 2, 3, 4), and 1D-polynitrogen ([N_4_]_∞_^*x*^^−^, *x* = 2–6) anions are able to accumulate different charges, one can expect that the N_6_ and N_8_ units can also exist in different charge states, and therefore can be found in other metal-nitrogen systems.

**Fig. 4:**
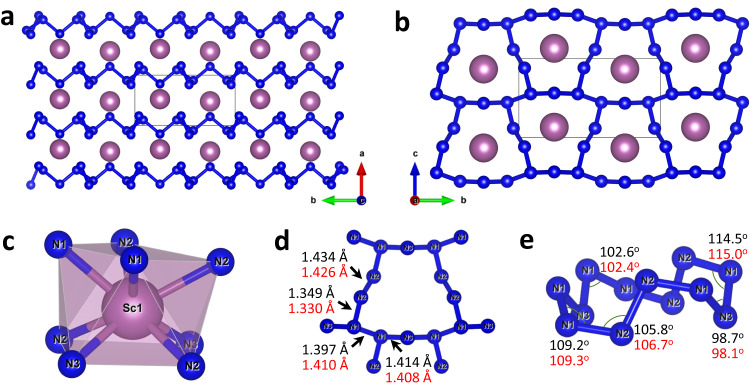
Crystal structure of ScN_5_ at 96 GPa. **a** A view of the crystal structure along the *c*-axis. **b** A view of the crystal structure along the *a*-axis. **c** The coordination environment of the Sc atom. **d** A specific view of N_12_ cycle along the *a*-axis. **e** A general view of N_12_ cycle. Sc atoms are purple, N atoms are blue; thin grey lines outline the unit cell. Values of bond lengths and angles obtained from the experiment are shown in black, while those obtained from the DFT calculations are shown in red.

The structure of ScN_5_ has the monoclinic space group *P*2_1_/*m* (#11) with one Sc and three N distinct atomic positions (see Supplementary Table 5 and the CIF for the full crystallographic data). Nitrogen atoms form corrugated 2D polymeric ${\!\,}_{\infty }{\!}^{2}({{{{{\rm{N}}}}}}_{5}^{3-})$ layers alternating along the *a*-axis built of fused N_12_ rings (Fig. 4a). Sc atoms are located in between the layers, in the way that the projection of Sc atoms along the *a*-axis is in the center of the N_12_ rings (Fig. 4b). Sc atoms are eight-fold coordinated (coordination number CN = 8, coordination polyhedron is a distorted square antiprism) by four N atoms of the lower layer and four N atoms of the upper layer (Fig. 4c).

The analysis of N-N lengths in ScN_5_ suggests that all N-N bonds are single bonds (Fig. 4d). All N atoms can be considered as *sp*^3^-hybridized, which also explains that the values of N-N-N angles in the N_12_ cycles are close to the ideal tetrahedra angle (98.7°−114.5°, Fig. 4e). N1 atoms make three covalent N-N bonds, while N2 and N3 atoms make only two, therefore one can suggest a −1 charge on the N2 and N3 atoms. It corresponds to the +3 oxidation state of Sc atoms.

Despite the theoretical prediction of four different structures with the ScN_5_ composition^39–41^, the here observed structure was not predicted. Usually in polynitrides nitrogen prefers to form 1D polymeric chains^20,28–33^, and among all the experimentally synthesized polynitrides up-to-date there is only one discovered polynitride with 2D polynitrogen layers—monoclinic BeN_4_^33^ with layers consisting of the fused N_10_ rings. The polynitrogen layers in ScN_5_ can be considered as distorted bp-N layers^2^, where 1/6 atoms are missing (Supplementary Fig. 4).

ScN_5_ is isostructural to a family of polyphosphides LnP_5_ (Ln=La-Lu, Y (except Eu and Pm)) known at ambient conditions^46,47^. It fully obeys the ninth high-pressure chemistry rule of thumb formulated in 1998: *“Elements behave at high pressures like the elements below them in the periodic table at lower pressures”* ^48^. The adoption of this structure type is also advantageous from a geometric point of view, since the ratio of ionic radii r(N^3^^−^)/r(Sc^3+^)=1.97 in ScN_5_ perfectly fits r(P^3^^−^)/r(Y^3+^) = 1.95 in the above-mentioned family member YP_5_.

Sc_4_N_3_ synthesized at 125 GPa has a well-known anti-Th_3_P_4_ structure type (space group *I*−43*d* (#220)) and contains only distinct, not-catenated N atoms (see Supplementary Table 6, Supplementary Fig. 5, and the CIF for the full crystallographic data), which we do not discuss in detail here. This Sc_4_N_3_ structure was predicted to be thermodynamically stable above 80 GPa^39^.

In order to get a deeper insight into the chemistry and the physical properties of the novel compounds, further DFT calculations were performed (see Methods section for details). As mentioned above, variable-cell structural relaxations for the Sc_2_N_6_, Sc_2_N_8_, and ScN_5_ compounds at the synthesis pressure closely reproduced structural parameters and bond lengths obtained from the experimental data. The phonon dispersion relations calculated in the harmonic approximation show that Sc_2_N_6_, Sc_2_N_8,_ and ScN_5_ phases are dynamically stable at 96 GPa and remain dynamically stable at ambient pressure (Supplementary Figs. 6–8). Considering dynamical stability at 1 bar, we have attempted to quench Sc_2_N_6_, Sc_2_N_8_, ScN_5_ phases, however, due to technical limitations of the decompression experiment (see footnote Supplementary Table 1), no conclusion regarding their recoverability could be made. To trace the structures’ behavior during the pressure release and to get the equations of state of all synthesized nitrogen-rich high-pressure scandium polynitrides, the full variable-cell structure relaxation for the Sc_2_N_6_, Sc_2_N_8_, and ScN_5_ compounds were performed with 10 GPa pressure steps in the range of 0–150 GPa (Supplementary Fig. 9). The volume-pressure dependences of DFT-relaxed structures of Sc_2_N_6_, Sc_2_N_8_, and ScN_5_ were fitted with a 3^rd^ order Birch-Murnaghan equation of state (Supplementary Fig. 10). The obtained bulk moduli (K_0_(Sc_2_N_6_) = 160 GPa, K_0_(Sc_2_N_8_) = 173 GPa, K_0_(ScN_5_) = 205 GPa) are lower than or comparable to the bulk modulus of known ScN (K_0_(ScN) = 207 GPa)^38^.

Under the same pressure, the volume per atom for all investigated nitrides monotonously linearly decreases with increasing nitrogen content (Supplementary Fig. 11a). Interestingly, the volume per nitrogen atom in the ScN-Sc_2_N_6_-Sc_2_N_8_-ScN_5_ series does not decrease with the degree of nitrogen polymerization (Supplementary Fig. 11b), so nitrogen polymerization probably is a way of crystal structure adaptation to closer N-N contacts.

While the structure of Sc_2_N_6_ has been predicted^39^, the crystal structures of Sc_2_N_8_ and ScN_5_ we observed have not been predicted. Remarkably, four different crystal structures with the ScN_5_ composition were proposed^39–41^, but the one we synthesized in the present study (*P*2_1_*/m* ScN_5_) was not among them. Our calculations of relative formation enthalpies of ScN_5_ for various predicted structures (*Cm* ScN_5_^39^, *P*−1 ScN_5_^39^, *C*2/*m* ScN_5_, ^40^ and *P*2_1_/*c* ScN_5_^41^) with respect to *P*2_1_*/m* ScN_5_ (Supplementary Fig. 12a) in the range of 0 to150 GPa have shown that above 46 GPa the *P*2_1_/*m* ScN_5_ phase is thermodynamically more stable than all other predicted phases. Below 46 GPa *P*−1 ScN_5_^39^ is more favorable. The *C*2*/m* ScN_5_^40^ and *P*2_1_*/c* ScN_5_^41^ phases are not energetically competitive with *P*2_1_*/m* ScN_5_ in the whole pressure range studied (Supplementary Fig. 12a).

To estimate the thermodynamic stability of the Sc_2_N_6_, Sc_2_N_8_, and ScN_5_ phases, the nitrogen-rich part of the static enthalpy convex hull was calculated at different pressures. Sc_2_N_6_ and ScN_5_ phases were found to be stable at the synthesis pressures (78 and 96 GPa, Supplementary Fig. 13a and Supplementary Fig. 12b), but Sc_2_N_8_ appears to be out of the convex hull (40 meV and 50 meV per atom above the convex hull at 78 and 96 GPa, respectively). Such insignificant departures from the convex hull, smaller than k_B_T at the synthesis temperature (2500 K, 215 meV), suggest that Sc_2_N_8_ may be thermodynamically stable at high temperatures and preserved as a metastable state under rapid T-quench to room temperature. ScN_5_ remains thermodynamically stable at least down to 40 GPa (Supplementary Fig. 13b), and Sc_2_N_6_—down to 30 GPa (Supplementary Fig. 13c), while at 20 GPa all nitrogen-rich scandium phases are out of the convex-hull (Supplementary Fig. 13d).

**Fig. 5:**
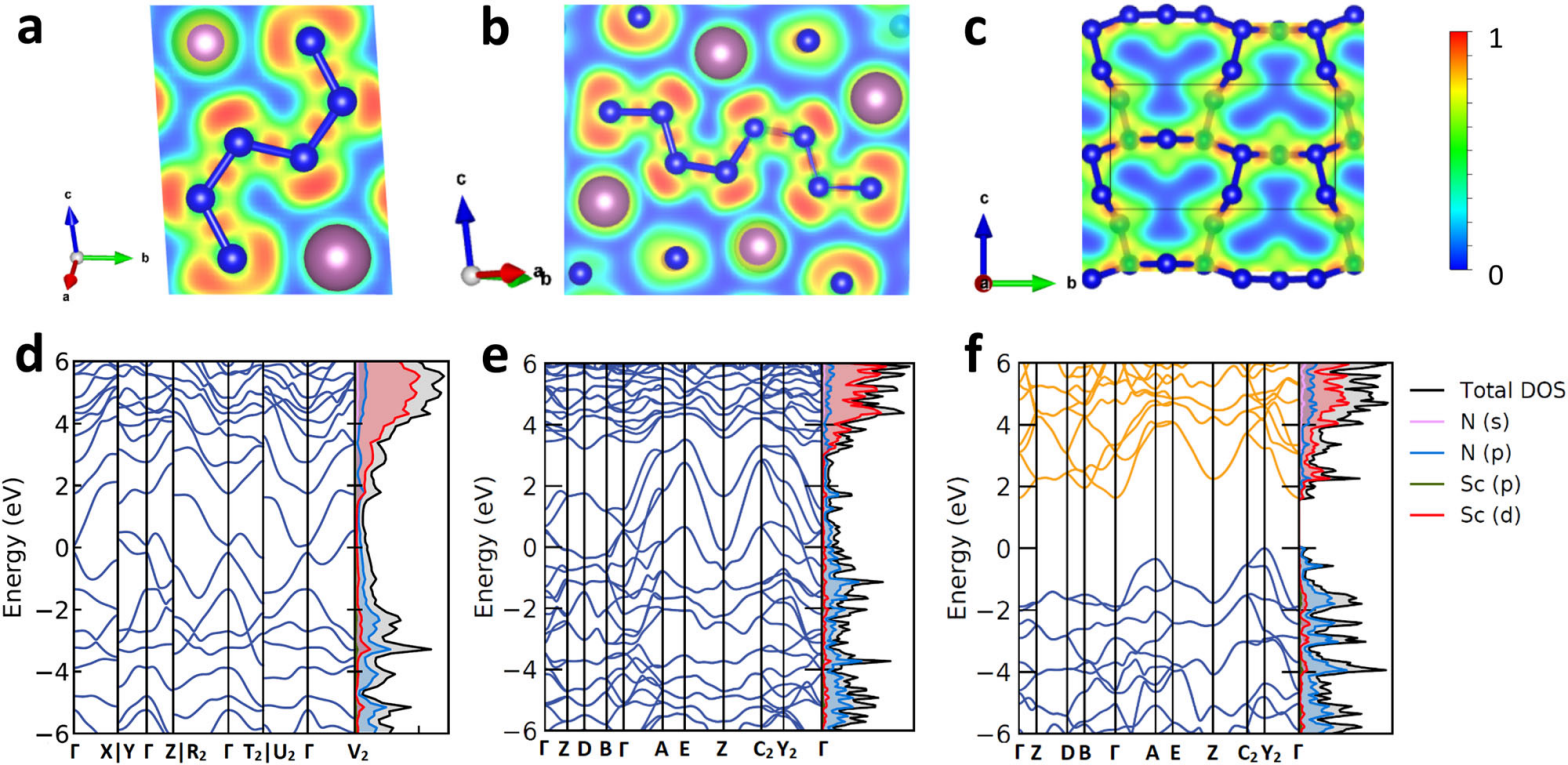
Calculated electronic properties of Sc_2_N_6_ at 78 GPa, and Sc_2_N_8,_ ScN_5_ at 96 GPa. Electron localization function calculated for (**a**) Sc_2_N_6_ in the (3 0 2) plane, (**b**) Sc_2_N_8_ in the (−2 4 1) plane, and (**c**) ScN_5_ in the (1 0 0) plane. The electron density of states of (**d**) Sc_2_N_6_, (**e**) Sc_2_N_8,_ and (**f**) ScN_5_.

The calculated electron localization functions for Sc_2_N_6_, Sc_2_N_8_, and ScN_5_ demonstrate a strong covalent bonding between nitrogen atoms within the N_6_, N_8_ units, and 2D-polynitrogen layers (Fig. 5a–c), while there is no covalent bonding between nitrogen and scandium atoms. The computed electron density of states (DOS) shows that Sc_2_N_6_ and Sc_2_N_8_ are metals (Fig. 5d, e) with an anion-driven metallicity^10^, since the main electronic contribution at the Fermi level comes from the nitrogen *p*-states. At the same time, ScN_5_ is an indirect semiconductor with a band gap of 1.8 eV at 96 GPa (Fig. 5f). One can explain such different electronic properties considering the chemical bonding in these compounds. In ScN_5_ there are only single N-N bonds, which means all π* antibonding nitrogen molecular orbitals are fully filled, whereas, in Sc_2_N_6_ and Sc_2_N_8_, containing delocalized π-bonds within N_6_^6^^−^ and N_8_^6^^−^ units, π* antibonding nitrogen states are partially filled and can conduct electrons through the π*-band. A similar trend of electronic properties with respect to the presence of N-N π-bonds is observed for many known polynitrides^27–33^. Among all known polynitrides there are only two compounds with solely σ N-N bonds: TaN_5_, which contains single-bonded branched polynitrogen chains^31^, and m-BeN_4_, which contains single-bonded 2D-polynitrogen layers^33^. Both compounds are semiconductors, as reported for TaN_5_^31^, and calculated for m-BeN_4_ in the present study (Supplementary Fig. 14). Other polynitrides contain N-N π-bonds and the majority of them (tr-BeN_4_, FeN_4_, α-ZnN_4_, β-ZnN_4_, TaN_4_, ReN_8_·xN_2_, WN_8_·N_2_, Os_5_N_28_·3N_2_, Hf_4_N_20_·N_2_, Hf_2_N_11_, Y_2_N_11_, YN_6_)^27–33^ exhibit an anion-driven metallicity.

Considering the dynamical stability of Sc_2_N_6_, Sc_2_N_8_, and ScN_5_ at ambient pressure, these phases might be preserved at ambient conditions as metastable and potentially can serve as high-energy-density materials. The key metrics of energetic materials’ performance^49^, such as volumetric and gravimetric energy densities, detonation velocity, and detonation pressure, were estimated for Sc_2_N_6_, Sc_2_N_8_, and ScN_5_ (Table 1) considering their decomposition to ScN and molecular nitrogen at 1 bar (see Methods section for details).

**Table 1:** Characteristics of Sc_2_N_6_, Sc_2_N_8_, ScN_5_ and TNT as energetic materials

Compound	Density *ρ*, g/cm^3^	Energy density		Detonation velocity *V*_d_, km/s	Detonation pressure *P*_d_, GPa
		gravimetric GED, kJ/g	volumetric VED, kJ/cm^3^		
Sc_2_N_6_	3.65	2.28	8.31	6.9	30
Sc_2_N_8_	3.58	3.07	11.0	8.3	43
ScN_5_	3.71	3.76	14.0	9.8	60
TNT	1.64^50^	4.3^51^	7.2^51^	6.9^50^	19^50^

The energy densities and explosive performance increase from Sc_2_N_6_ to ScN_5_ along with the increase in nitrogen content. Due to the higher density of scandium nitrides compared to organic explosives, they possess extremely high volumetric energy densities that are higher than the typical energy density of TNT. The estimated gravimetric energy densities are lower than that of TNT, but higher than those of many other polynitrides^31^ since scandium is a light metal. The estimated detonation velocity and detonation pressure of scandium polynitrides are also higher than those of TNT. Thus, the Sc_2_N_6_, Sc_2_N_8_, and ScN_5_ are promising high-energy-density materials.

To summarize, in this study, four novel Sc-N phases—Sc_2_N_6_, Sc_2_N_8_, ScN_5_, and Sc_4_N_3_—were synthesized from Sc and N_2_ by laser-heating at 2500 K at pressures between 78 and 125 GPa. Nitrogen-rich scandium polynitrides Sc_2_N_6_, Sc_2_N_8_, and ScN_5_ demonstrate a unique nitrogen catenation: they feature N_6_ units, N_8_ units, and 2D polynitrogen ${\!\,}_{\infty }{\!\,}^{2}({{{{{\rm{N}}}}}}_{5}^{3-})$ layers consisting of N_12_ fused rings, respectively. DFT calculations showed that all three scandium polynitrides are dynamically stable at the synthesis pressure as well as at 1 bar. Sc_2_N_6_ and Sc_2_N_8_ are metals with the main electronic contribution at the Fermi level that comes from the nitrogen *p*-states, while ScN_5_ is an indirect semiconductor. Synthesized Sc_2_N_6_, Sc_2_N_8_, and ScN_5_ compounds are promising high-energy-density materials with volumetric energy densities, detonation velocities, and detonation pressures higher than those of TNT.

One can expect that the N_6_ and N_8_ units will be stabilized at ambient conditions in the future, considering a positive example of CsN_5_ high-pressure synthesis and subsequent stabilization of the N_5_^−^ anion at atmospheric pressure. It may not only open access to novel high-energy-density materials but also to analogues of Li- and Mg- metalorganic compounds that are currently widely used in organic synthesis. N_6_ and N_8_ units, if used as building blocks in organic chemistry, may provide new routes for the targeted synthesis of novel N-heteroatomic organic, metalorganic, and coordination compounds.

## Methods

### Sample preparation

The BX90-type large X-ray aperture DACs^52^ equipped with Boehler-Almax type diamonds^53^ (culet diameters are 250, 120, and 80 μm) were used in the experiments. The sample chambers were formed by pre-indenting of rhenium gaskets to 20, 18, and 15 μm thickness and laser-drilling a hole of 115, 60 and 40 μm, respectively, in diameter in the center of the indentation. A DAC equipped with 250-μm culet anvils was used for the experiment at 50(1) GPa; a DAC equipped with 120-μm culet anvils was used for experiments at 78(2) and 96(2); and a DAC equipped with 80-μm culet anvils was used for the experiment at 125(2) GPa. A piece of scandium (99.9%, Sigma Aldrich) was placed in a sample chamber, then molecular nitrogen (purity grade N5.0) was loaded using a BGI high-pressure gas loading system (1300 bars)^54^. The sizes of the scandium pieces were 40 × 40 × 8 μm^3^ for 250 μm culet anvils and not bigger than 15 × 15 × 5 μm^3^ for DACs with anvils of all other sizes. The samples were compressed to target pressure (50(1), 78(2), 96(2), and 125(2) GPa) and then laser-heated up to 2500(200) K using a home-made double-sided laser-heating system equipped with two YAG lasers (*λ* = 1064 nm) and the IsoPlane SCT 320 spectrometer with a 1024 × 2560 PI-MAX 4 camera for the collection of thermal emission spectra from the heated spot^55^. The temperature during the laser heating was determined by fitting of sample’s thermal emission spectra to the grey body approximation of Planck’s radiation function in a given wavelength range (570–830 nm). The pressure in the DACs was determined using the Raman signal from the diamond anvils^56^ and monitored additionally by X-ray diffraction of the Re gasket edge using the rhenium equation of state^57^.

### X-ray diffraction

The X-ray diffraction studies were done at the ID11 beamline (*λ* = 0.2843 Å and *λ* = 0.2846 Å) and ID15b beamline (*λ* = 0.4100 Å) of the Extreme Brilliant Source European Synchrotron Radiation Facility (EBS-ESRF) as well as at the GSECARS 13IDD beamline of the APS (*λ* = 0.2952 Å). At ID11 beamline of ESRF the X-ray beam was focused down to 0.75 × 0.75 μm^2^ and data was collected with Eiger2X CdTe 4 M hybrid photon counting pixel detector. At ID15b beamline of ESRF the X-ray beam was focused down to 1.5 × 1.5 μm^2^ and data was collected with Eiger2X CdTe 9 M hybrid photon counting pixel detector. At 13IDD beamline of APS the X-ray beam was focused down to 2 × 2 μm^2^ and data was collected with Pilatus 1 M detector. In order to determine the position of the polycrystalline sample on which the single-crystal X-ray diffraction acquisition is obtained, a full X-ray diffraction mapping of the pressure chamber was achieved. The sample position displaying the most and the strongest single-crystal reflections belonging to the phase of interest was chosen for the collection of single-crystal data, collected in step-scans of 0.5° from −36° to +36°. The CrysAlis^Pro^ software package^58^ was used for the analysis of the single-crystal XRD data (peak hunting, indexing, data integration, frame scaling, and absorption correction). To calibrate an instrumental model in the CrysAlis^Pro^ software, i.e., the sample-to-detector distance, detector’s origin, offsets of the goniometer angles, and rotation of both the X-ray beam and detector around the instrument axis, we used a single crystal of orthoenstatite [(Mg_1.93_Fe_0.06_)(Si_1.93_,Al_0.06_)O_6_, *Pbca* space group, *a* = 8.8117(2) Å, *b* = 5.18320(10) Å, and *c* = 18.2391(3) Å]. The DAFi program was used for the search of reflection’s groups belonging to the individual single crystal domains^59^. Using the OLEX2 software package^60^, the structures were solved with the ShelXT structure solution program^61^ using intrinsic phasing and refined with the ShelXL^62^ refinement package using least-squares minimization. Crystal structure visualization was made with the VESTA software^63^.

### Theoretical calculations

First-principles calculations were performed using the framework of density functional theory (DFT) as implemented in the Vienna Ab initio Simulation Package (VASP)^64^. The Projector-Augmented-Wave (PAW) method^65^ was used to expand the electronic wave function in plane waves. The Generalized Gradient Approximation (GGA) functional is used for calculating the exchange-correlation energies, as proposed by Perdew–Burke–Ernzerhof (PBE)^66^. The recommended PAW potentials “Sc_sv” and “N” with the following valence configurations of 3*s*^2^3*p*^6^4*s*^2^3*d*^1^ for Sc and 2*s*^2^2*p*^3^ for N were used. We used the Monkhorst–Pack scheme with 10 × 10 × 10 for ScN, 12 × 8 × 8 for Sc_2_N_6_, 10 × 6 × 4 for Sc_2_N_8_, 12 × 6 × 12 for ScN_5_ *k*-points for Brillouin zone sampling, and the plane-wave kinetic energy cutoff was set to 800 eV, with which total energies are converged to better than 2 meV/atom. The electronic convergence criterion was set to Δ*E* = 10^−8^ eV, this minimized the interatomic forces to *F*_atom_ < 10^−3^ eV/Å. For electron band structure calculations the 1.5−2 fold denser *k*-points grids were used. The finite displacement method, as implemented in PHONOPY^67^, was used to calculate phonon frequencies and phonon band structures. The 4×3×3, 3×2×2, and 3 × 2 × 3 supercells with 4 × 4 × 4 *k*-points grids for Sc_2_N_6_, Sc_2_N_8_, and ScN_5_, respectively, were used for phonon calculations and displacement amplitudes were of 0.01 Å.

The gravimetric and volumetric energy densities of Sc_2_N_6_, Sc_2_N_8_, and ScN_5_ were calculated considering the enthalpy change ΔH for the following chemical decomposition reactions at ambient pressure at 0 K (the *Fm*−3*m*-ScN and *α*-N_2_ structures of products were used in the calculations since they are the most stable polymorphs at such conditions):

$${{{{{{\rm{Sc}}}}}}}_{2}{{{{{{\rm{N}}}}}}}_{6}\to 2\,{{{{{\rm{ScN}}}}}}+2\,{{{{{{\rm{N}}}}}}}_{2}$$

$${{{{{{\rm{Sc}}}}}}}_{2}{{{{{{\rm{N}}}}}}}_{8}\to 2\,{{{{{\rm{ScN}}}}}}+3\,{{{{{{\rm{N}}}}}}}_{2}$$

$${{{{{{\rm{ScN}}}}}}}_{5}\to {{{{{\rm{ScN}}}}}}+2\,{{{{{{\rm{N}}}}}}}_{2}$$

The detonation velocity (*V*_*d*_, km/s) and detonation pressure (*P*_*d*_, GPa) of the Sc_2_N_6_, Sc_2_N_8_, and ScN_5_ were estimated by the Kamlet-Jacobs empirical equations^50^:

1 $${V}_{d}={(N{{\cdot }}{M}^{0.5}{{\cdot }}{{GED}}^{0.5})}^{0.5}{{\cdot }}(1.011+1.312\rho )$$

2 $${P}_{d}=1.588{{\cdot }}N{{\cdot }}{M}^{0.5}{{\cdot }}{{GED}}^{0.5}{{\cdot }}{\rho }^{2}$$

where *N* is the number of moles of gaseous detonation product (nitrogen gas) per gram of explosive, *M* is the molar mass (28 g/mol) of nitrogen gas, *GED* is gravimetric energy density in cal/g, and *ρ* is density in g/cm^3^.

## Supplementary information

Supporting Information

Peer Review File

## Source data

Source Data

## Data Availability

The full crystallographic data for structures reported in this article have been deposited at the Inorganic Crystal Structure Database (ICSD) under deposition numbers CSD 2252030−2252036. These data can be obtained from CCDC’s and FIZ Karlsruhe’s free service for viewing and retrieving structures (https://www.ccdc.cam.ac.uk/structures/). The crystallographic information (CIF files, FCF files, and the corresponding CheckCIF reports) is also available as Source data. All other datasets generated and/or analyzed during the current study are available from the corresponding author upon request. Source data are provided with this paper.
