# Supplementary material for: Stabilization of N6 and N8 anionic units and 2D polynitrogen layers in high-pressure scandium polynitrides
Source: Nat Commun. 2024 Mar 12;15:2244. doi: 10.1038/s41467-024-46313-9 (PMC11636835; doi:10.1038/s41467-024-46313-9)
Supplement: Supplementary file 1 — Supporting Information [file 41467_2024_46313_MOESM1_ESM.pdf]

Supporting Information

Stabilization of N_6 and N_8 anionic units and 2D polynitrogen layers in high-pressure scandium polynitrides

Andrey Aslandukov[1,2*], Alena Aslandukova[1], Dominique Laniel[3], Saiana Khandarkhaeva[1], Yuqing Yin[2], Fariia I. Akbar[1], Stella Chariton[4], Vitali Prakapenka[4], Eleanor Lawrence Bright[5], Carlotta Giacobbe[5], Jonathan Wright[5], Davide Comboni[5], Michael Hanfland[5], Natalia Dubrovinskaia[2,6] Leonid Dubrovinsky[1]

[1] Bavarian Research Institute of Experimental Geochemistry and Geophysics (BGI), University of Bayreuth, Universitaetstrasse 30, 95440 Bayreuth, Germany

[2] Material Physics and Technology at Extreme Conditions, Laboratory of Crystallography, University of Bayreuth, 95440 Bayreuth, Germany

[3] Centre for Science at Extreme Conditions and School of Physics and Astronomy, University of Edinburgh, EH9 3FD Edinburgh, United Kingdom

[4] Center for Advanced Radiation Sources, University of Chicago, Chicago, Illinois 60637, USA

[5] European Synchrotron Radiation Facility, BP 220, 38043 Grenoble Cedex, France

[6] Department of Physics, Chemistry and Biology (IFM), Linköping University, SE-581 83, Linköping, Sweden

*Correspondence to andrii.aslandukov@uni-bayreuth.de

Supplementary Tables

Supplementary Table 1. List of the samples investigated in the present study

<table>
<tr><th rowspan="2">Sample</th><th rowspan="2">Culet size, µm</th><th rowspan="2">Reaction mixture</th><th rowspan="2">Temperature, K</th><th colspan="2">Pressure, GPa</th><th rowspan="2">Reaction products</th><th rowspan="2">Attempt to decompress</th></tr>
<tr><th>Raman diamond edge</th><th>Re EoS</th></tr>
<tr><td>DAC#1</td><td>250</td><td>Sc + N_2</td><td>2500(200)</td><td>50(1)</td><td>50(1)</td><td>ScN</td><td>No</td></tr>
<tr><td rowspan="2">DAC#2[1]</td><td rowspan="2">120</td><td rowspan="2">Sc + N_2</td><td>2500(200)</td><td>78(2)</td><td>79(2)</td><td>ScN, Sc_2N_6, Sc_2N_8</td><td rowspan="2">Yes[2]</td></tr>
<tr><td>2500(200)</td><td>96(2)</td><td>98(2)</td><td>ScN, Sc_2N_8, ScN_5</td></tr>
<tr><td>DAC#3</td><td>80</td><td>Sc + N_2</td><td>2500(200)</td><td>125(2)</td><td>125(2)</td><td>Sc_4N_3, ScN_5</td><td>No</td></tr>
</table>

[1]Two scandium pieces were loaded into the sample chamber of DAC#2. One of the pieces was laser heated at 96(2) GPa, while another one at 78(2) GPa.

[2]In order to check the recoverability of Sc_2N_6, Sc_2N_8, ScN_5 phases DAC#2 was decompressed. Unfortunately, at 50(2) GPa nitrogen pressure transmitting medium went out through microcracks in the diamond resulting in the complete gasket closure and dropping pressure to 1 atm. The sample could not be found afterward, therefore no conclusion regarding the recoverability of Sc_2N_6, Sc_2N_8, and ScN_5 can be made. Unfortunately, it is a technical limitation of the decompression experiment started from the pressures above ~70 GPa in a gas pressure transmitting medium. It is a typical behavior of the culets (*i.e.* 120 µm or smaller) of double-bevel diamonds: above ~70 GPa the square-like cracks appear around the culet, and during the decompression in a gas-pressure transmitting medium diffuses into the crack resulting in a crack expansion and cell depressurization.

Supplementary Table 2. Structure refinement details of ScN at 50 GPa. The full crystallographic data was deposited to the ICSD under the deposition number CSD 2252036. The A and B alerts revealed by CheckCIF are listed and explained after the table.

<table>
<tr><td colspan="3">Chemical formula</td><td colspan="3">ScN</td></tr>
<tr><td colspan="3">Temperature (K)</td><td colspan="3">293</td></tr>
<tr><td colspan="3">Pressure (GPa)</td><td colspan="3">50(2)</td></tr>
<tr><td colspan="6">Crystal data</td></tr>
<tr><td colspan="3">Mr</td><td colspan="3">58.97</td></tr>
<tr><td colspan="3">ρ (g/cm^3)</td><td colspan="3">5.105</td></tr>
<tr><td colspan="3">Crystal system, space group</td><td colspan="3">cubic, Fm-3m</td></tr>
<tr><td colspan="3">a (Å)</td><td colspan="3">4.2492(7)</td></tr>
<tr><td colspan="3">V (Å^3)</td><td colspan="3">76.72(4)</td></tr>
<tr><td colspan="3">Z</td><td colspan="3">4</td></tr>
<tr><td colspan="3">Radiation type</td><td colspan="3">X-ray, λ = 0.29521 Å</td></tr>
<tr><td colspan="3">μ (mm^{-1})</td><td colspan="3">0.709</td></tr>
<tr><td colspan="6">Data collection</td></tr>
<tr><td colspan="3">No. of measured, independent and observed [I > 2σ(I)] reflections</td><td colspan="3">40/19/19</td></tr>
<tr><td colspan="3">R_{int}</td><td colspan="3">6.88%</td></tr>
<tr><td colspan="3">(sin $\theta/\lambda)_{max}$ (Å^{-1})</td><td colspan="3">0.849</td></tr>
<tr><td colspan="6">Refinement</td></tr>
<tr><td colspan="3">R[F^2 > 4σ(F^2)], wR(F^2), GOF</td><td colspan="3">3.41%, 7.72%, 1.202</td></tr>
<tr><td colspan="3">data/parameters ratio</td><td colspan="3">19/3</td></tr>
<tr><td colspan="3">$\Delta\rho_{max}$, $\Delta\rho_{min}$ (e Å^{-3})</td><td colspan="3">0.630, -1.025</td></tr>
<tr><td colspan="6">Atomic positions and equivalent isotropic ADPs</td></tr>
<tr><td rowspan="2">Atom</td><td rowspan="2">Wyckoff site</td><td colspan="3">Fractional atomic coordinates</td><td rowspan="2">U_{eq} (Å^2)</td></tr>
<tr><td>x</td><td>y</td><td>z</td></tr>
<tr><td>Sc1</td><td>4a</td><td>0</td><td>0</td><td>0</td><td>U_{eq} = 0.0070(7)</td></tr>
<tr><td>N1</td><td>4b</td><td>0.5</td><td>0.5</td><td>0.5</td><td>U_{eq} = 0.0051(14)</td></tr>
</table>

Datablock: ScN_50GPa

PLAT088_ALERT_3_B Poor Data / Parameter Ratio .... 6.33 Note

- Author Response: This measurement was performed at high pressure. The diamond anvil cell metallic body typically shadows more than 60% of the reflections.

PLAT113_ALERT_2_B ADDSYM Suggests Possible Pseudo/New Space Group Pm-3m Check

- Author Response: Fm-3m spacegroup is correct.

Supplementary Table 3. Structure refinement details of Sc_2N_6 at 78 GPa. The full crystallographic data was deposited to the ICSD under the deposition number CSD 2252033. The A and B alerts revealed by CheckCIF are listed and explained after the table.

Chemical formula	Sc_2N_6
Temperature (K)	293
Pressure (GPa)	78(2)
**Crystal data**	
Mr	173.98
ρ (g/cm^3)	5.092
Crystal system, space group	triclinic, *P*-1
a (Å)	2.9170(3)
b (Å)	4.3283(10)
c (Å)	4.812(2)
α (°)	99.36(3)
β (°)	104.29(2)
γ (°)	99.517(15)
V (Å^3)	56.73(3)
Z	1
Radiation type	X-ray, λ = 0.2846 Å
μ (mm^{-1})	0.471
**Data collection**	
No. of measured, independent and observed [I > 2σ(I)] reflections	302/224/213
R_{int}	0.25%
$(\sin θ/λ)_{max}$ (Å^{-1})	1.121
**Refinement**	
R[$F^2 > 4σ(F^2)$], wR(F^2), GOF	4.12%, 12.34%, 1.188
data/parameters ratio	224/22
$Δρ_{max}$, $Δρ_{min}$ (e Å^{-3})	0.564, -0.707

Atomic positions and equivalent isotropic (or isotropic) ADPs

Atom	Wyckoff site	Fractional atomic coordinates			U_{iso} or U_{eq} (Å^2)
		x	y	z	
Sc1	2*i*	0.41540(12)	0.79589(14)	0.1946(2)	U_{eq} = 0.0054(7)
N1	2*i*	0.2595(7)	0.2909(8)	0.1278(14)	U_{iso} = 0.0072(3)
N2	2*i*	0.1124(7)	0.1685(9)	0.3293(14)	U_{iso} = 0.0083(4)
N3	2*i*	0.0020(7)	0.3614(8)	0.5348(13)	U_{iso} = 0.0074(4)

Datablock: Sc_2N_6_78GPa

PLAT029_ALERT_3_A _diffrn_measured_fraction_theta_full value Low . 0.293 Why?

- Author Response: This measurement was performed at high pressure which, due to the high pressure apparatus, limits the theta range. Indeed, the diamond anvil cell metallic body typically shadows more than 60% of the reflections.

PLAT911_ALERT_3_B Missing FCF Refl Between Thmin & STh/L= 0.600 143 Report

- Author Response: This measurement was performed at high pressure which, due to the high pressure apparatus, limits the theta range. Indeed, the diamond anvil cell metallic body typically shadows more than 60% of the reflections.

Supplementary Table 4. Structure refinement details of Sc_2N_8 at 78 and 96 GPa. The full crystallographic data were deposited to the ICSD under the deposition numbers CSD 2252034 and CSD 2252035. The A and B alerts revealed by CheckCIF are listed and explained after the table.

Chemical formula	Sc_2N_8	Sc_2N_8
Temperature (K)	293	293
Pressure (GPa)	78(2)	96(2)
**Crystal data**		
Mr	202.0	202.0
ρ (g/cm^3)	4.857	4.994
Crystal system, space group	monoclinic, $P2_1/c$	monoclinic, $P2_1/c$
a (Å)	3.3278(6)	3.2884(16)
b (Å)	5.6802(8)	5.6390(9)
c (Å)	7.3964(4)	7.342(2)
β (°)	98.905(9)	99.36(4)
V (Å^3)	138.13(3)	134.32(8)
Z	2	2
Radiation type	X-ray, λ = 0.2846 Å	X-ray, λ = 0.2843 Å
μ (mm^{-1})	0.401	0.411
**Data collection**		
No. of measured, independent and observed [I > 2σ(I)] reflections	946/575/429	388/320/215
R_{int}	2.40%	2.01%
$(\sin \theta/\lambda)_{max}$ (Å^{-1})	1.161	1.045
**Refinement**		
R[F^2 > 4σ(F^2)], wR(F^2), GOF	4.38%, 11.78%, 1.074	4.11%, 11.07%, 1.009
data/parameters ratio	575/46	320/26
$\Delta\rho_{max}$, $\Delta\rho_{min}$ (e Å^{-3})	1.147, -0.722	0.552, -0.584

Atomic positions and equivalent isotropic ADPs at 78(2) GPa

Atom	Wyckoff site	Fractional atomic coordinates			U_{eq} (Å^2)
		x	y	z	
Sc1	4*e*	0.1326(2)	0.87943(11)	0.32610(6)	U_{eq} = 0.00742(15)
N1	4*e*	0.3159(11)	0.6093(5)	0.1307(3)	U_{eq} = 0.0087(5)
N2	4*e*	0.3777(10)	0.2554(5)	0.3724(3)	U_{eq} = 0.0077(5)
N3	4*e*	0.3769(10)	0.1903(5)	0.0434(3)	U_{eq} = 0.0078(5)
N4	4*e*	0.0859(10)	0.0525(5)	0.0761(3)	U_{eq} = 0.0077(5)

Atomic positions and equivalent isotropic (or isotropic) ADPs at 96(2) GPa

Atom	Wyckoff site	Fractional atomic coordinates			U_{iso} or U_{eq} (Å^2)
		x	y	z	
Sc1	4*e*	0.1314(3)	0.88020(12)	0.32608(14)	U_{eq} = 0.0084(3)
N1	4*e*	0.3111(15)	0.6111(6)	0.1312(6)	U_{iso} = 0.0100(6)
N2	4*e*	0.3781(17)	0.2583(5)	0.3726(6)	U_{iso} = 0.0096(6)
N3	4*e*	0.3759(16)	0.1906(5)	0.0444(5)	U_{iso} = 0.0090(6)
N4	4*e*	0.0891(17)	0.0513(6)	0.0773(6)	U_{iso} = 0.0099(6)

Datablock: Sc_2N_8_78GPa

PLAT029_ALERT_3_A _diffrn_measured_fraction_theta_full value Low . 0.545 Why?

- Author Response: This measurement was performed at high pressure which, due to the high pressure apparatus, limits the theta range. Indeed, the diamond anvil cell metallic body typically shadows more than 60% of the reflections.

PLAT911_ALERT_3_B Missing FCF Refl Between Thmin & STh/L= 0.600 111 Report

- Author Response: This measurement was performed at high pressure which, due to the high pressure apparatus, limits the theta range. Indeed, the diamond anvil cell metallic body typically shadows more than 60% of the reflections.

Datablock: Sc_2N_8_96GPa

PLAT029_ALERT_3_A _diffrn_measured_fraction_theta_full value Low . 0.407 Why?

- Author Response: This measurement was performed at high pressure which, due to the high pressure apparatus, limits the theta range. Indeed, the diamond anvil cell metallic body typically shadows more than 60% of the reflections.

PLAT911_ALERT_3_B Missing FCF Refl Between Thmin & STh/L= 0.600 144 Report

- Author Response: This measurement was performed at high pressure which, due to the high pressure apparatus, limits the theta range. Indeed, the diamond anvil cell metallic body typically shadows more than 60% of the reflections.

Supplementary Table 5. Structure refinement details of ScN_5 at 96 and 125 GPa. The full crystallographic data were deposited to the ICSD under the deposition numbers CSD 2252030 and CSD 2252031. The A and B alerts revealed by CheckCIF are listed and explained after the table.

Chemical formula	ScN_5	ScN_5
Temperature (K)	293	293
Pressure (GPa)	96(2)	125(2)
**Crystal data**		
Mr	115.01	115.01
ρ (g/cm^3)	4.971	5.282
Crystal system, space group	monoclinic, $P2_1/m$	monoclinic, $P2_1/m$
a (Å)	3.3225(6)	3.203(5)
b (Å)	6.440(3)	6.3576(8)
c (Å)	3.7067(4)	3.6943(6)
β (°)	104.339(13)	105.99(6)
V (Å^3)	76.84(4)	72.31(12)
Z	2	2
Radiation type	X-ray, λ = 0.2843 Å	X-ray, λ = 0.4100 Å
μ (mm^{-1})	0.373	0.373
**Data collection**		
No. of measured, independent and observed [I > 2σ(I)] reflections	446/264/202	172/106/98
R_{int}	3.21%	2.02%
$(\sin θ/λ)_{max}$ (Å^{-1})	1.067	0.876
**Refinement**		
R[F^2 > 4σ(F^2)], wR(F^2), GOF	3.63%, 8.77%, 1.008	4.43%, 11.96%, 1.154
data/parameters ratio	264/31	106/15
$Δρ_{max}$, $Δρ_{min}$ (e Å^{-3})	0.724, -0.789	0.605, -0.668

Atomic positions and equivalent isotropic ADPs at 96(2) GPa

Atom	Wyckoff site	Fractional atomic coordinates			U_{eq} (Å^2)
		x	y	z	
Sc1	2*e*	0.4779(2)	0.25	0.13202(18)	U_{eq} = 0.0076(3)
N1	4*f*	0.1061(6)	0.5835(8)	0.4462(5)	U_{eq} = 0.0082(9)
N2	4*f*	0.1897(7)	0.5396(8)	0.1165(5)	U_{eq} = 0.0099(10)
N3	2*e*	0.1740(10)	0.25	0.5651(8)	U_{eq} = 0.0078(13)

Atomic positions and isotropic ADPs at 125(2) GPa

Atom	Wyckoff site	Fractional atomic coordinates			U_{iso} (Å^2)
		x	y	z	
Sc1	2*e*	0.4623(11)	0.25	0.1205(3)	U_{iso} = 0.0102(4)
N1	4*f*	0.102(4)	0.5853(6)	0.4492(11)	U_{iso} = 0.0106(8)
N2	4*f*	0.187(4)	0.5457(6)	0.1196(10)	U_{iso} = 0.0109(8)
N3	2*e*	0.184(5)	0.25	0.5551(15)	U_{iso} = 0.0097(10)

Datablock: ScN_5_96GPa

PLAT029_ALERT_3_A _diffrn_measured_fraction_theta_full value Low . 0.547 Why?

- Author Response: This measurement was performed at high pressure which, due to the high pressure apparatus, limits the theta range. Indeed, the diamond anvil cell metallic body typically shadows more than 60% of the reflections.

PLAT911_ALERT_3_B Missing FCF Refl Between Thmin & STh/L= 0.600 68 Report

- Author Response: This measurement was performed at high pressure which, due to the high pressure apparatus, limits the theta range. Indeed, the diamond anvil cell metallic body typically shadows more than 60% of the reflections.

Datablock: ScN_5_125GPa

ATOM007_ALERT_1_A _atom_site_aniso_label is missing Unique label identifying the atom site.

- Author Response: Due to poor data/parameter ratio it is not possible to refine atoms in the anisotropic approximation.

PLAT029_ALERT_3_A _diffrn_measured_fraction_theta_full value Low . 0.392 Why?

- Author Response: This measurement was performed at high pressure which, due to the high pressure apparatus, limits the theta range. Indeed, the diamond anvil cell metallic body typically shadows more than 60% of the reflections.

PLAT088_ALERT_3_B Poor Data / Parameter Ratio .... 7.07 Note

- Author Response: Low number of reflections due to low quality of the crystallite under higher-than-megabar pressure and due to limitations of the high pressure apparatus.

PLAT149_ALERT_3_B s.u. on the beta Angle is Too Large .... 0.06 Degree

- Author Response: The precision of beta angle determination is a bit lower than usual, because of insufficient statistics. The reason is the low number of reflections, especially at high angles, due to low quality of the crystallite under higher-than-megabar pressure and due to limitations of the high pressure apparatus.

PLAT911_ALERT_3_B Missing FCF Refl Between Thmin & STh/L= 0.600 64 Report

- Author Response: This measurement was performed at high pressure which, due to the high pressure apparatus, limits the theta range. Indeed, the diamond anvil cell metallic body typically shadows more than 60% of the reflections.

Supplementary Table 6. Structure refinement details of Sc_4N_3 at 125 GPa. The full crystallographic data was deposited to the ICSD under the deposition number CSD 2252032. No A or B alerts were revealed by CheckCIF.

Chemical formula			Sc_4N_3		
Temperature (K)			293		
Pressure (GPa)			125(2)		
**Crystal data**					
Mr			221.87		
ρ (g/cm^3)			6.301		
Crystal system, space group			cubic, *I*-43*d*		
a (Å)			6.1613(13)		
V (Å^3)			233.89(15)		
Z			4		
Radiation type			X-ray, λ = 0.4100 Å		
μ (mm^{-1})			2.232		
**Data collection**					
No. of measured, independent and observed [I > 2σ(I)] reflections			246/83/80		
R_{int}			2.55%		
$(\sin \theta/\lambda)_{max}$ (Å^{-1})			0.8740		
**Refinement**					
$R[F^2 > 4\sigma(F^2)]$, $wR(F^2)$, GOF			2.66%, 6.37%, 1.078		
data/parameters ratio			83/6		
$\Delta\rho_{max}$, $\Delta\rho_{min}$ (e Å^{-3})			0.481, -0.534		
**Atomic positions and equivalent isotropic ADPs**					
Atom	Wyckoff site	Fractional atomic coordinates			U_{eq} (Å^2)
		x	y	z	
Sc1	16*c*	0.06784(8)	0.06784(8)	0.06784(8)	U_{eq} = 0.0080(3)
N1	12*a*	0	0.25	0.375	U_{eq} = 0.0095(11)

Supplementary Table 7. Experimentally determined crystallographic data for Sc_2N_6 at 78 GPa in comparison with the corresponding DFT-relaxed structure. Note that pressure was fixed in theoretical simulations, while volume of the unit cell, lattice parameters and equilibrium state parameters were calculated.

	Exp.			Calc.		
**Space group**	*P*-1			*P*-1		
**Volume**	56.73(3) Å^3			57.25 Å^3		
**Lattice parameters**	a = 2.9170(3) Å<br>b = 4.3283(10) Å<br>c = 4.812(2) Å<br>α = 99.36(3)°<br>β = 104.29(2)°<br>γ = 99.517(15)°			a = 2.9135 Å<br>b = 4.3291 Å<br>c = 4.8517 Å<br>α = 99.04°<br>β = 104.02°<br>γ = 99.731°		
**Atomic positions**	Sc1	x	0.41540(12)	Sc1	x	0.41668
		y	0.79589(14)		y	0.79662
		z	0.1946(2)		z	0.19583
	N1	x	0.2595(7)	N1	x	0.25922
		y	0.2909(8)		y	0.29398
		z	0.1278(14)		z	0.12654
	N2	x	0.1124(7)	N2	x	0.10949
		y	0.1685(9)		y	0.16759
		z	0.3293(14)		z	0.32841
	N3	x	0.0020(7)	N3	x	0.00298
		y	0.3614(8)		y	0.36034
		z	0.5348(13)		z	0.53232

Supplementary Table 8. Experimentally determined crystallographic data for Sc_2N_8 at 96 GPa in comparison with the corresponding DFT-relaxed structure. Note that pressure was fixed in theoretical simulations, while volume of the unit cell, lattice parameters and equilibrium state parameters were calculated.

	Exp.			Calc.		
**Space group**	$P2_1/c$			$P2_1/c$		
**Volume**	134.32(8)Å^3			133.59 Å^3		
**Lattice parameters**	a = 3.2884(16) Å<br>b = 5.6390(9) Å<br>c = 7.342(2) Å<br>β = 99.36°			a = 3.3081 Å<br>b = 5.5730 Å<br>c = 7.3612 Å<br>β = 100.14°		
**Atomic positions**	Sc1	x	0.1314(3)	Sc1	x	0.1291
		y	0.8802(1)		y	0.8826
		z	0.3261(1)		z	0.3270
	N1	x	0.3111(15)	N1	x	0.3159
		y	0.6111(6)		y	0.6148
		z	0.1312(6)		z	0.1332
	N2	x	0.3781(17)	N2	x	0.3754
		y	0.2583(5)		y	0.2576
		z	0.3726(6)		z	0.3723
	N3	x	0.3759(16)	N3	x	0.3775
		y	0.1906(5)		y	0.1905
		z	0.0444(5)		z	0.0441
	N4	x	0.0891(17)	N4	x	0.0855
		y	0.0513(6)		y	0.0545
		z	0.0773(6)		z	0.0755

Supplementary Table 9. Experimentally determined crystallographic data for ScN_5 phase at 96 GPa in comparison with the corresponding DFT-relaxed structure. Note that pressure was fixed in theoretical simulations, while volumes of the unit cells, lattice parameters and equilibrium state parameters were calculated.

	Exp.			Calc.		
**Space group**	*P*2$_1$/*m*			*P*2$_1$/*m*		
**Volume**	76.84(4) Å^3			76.53 Å^3		
**Lattice parameters**	a = 3.3225(6) Å<br>b = 6.440(3) Å<br>c = 3.7067(4) Å<br>β = 104.339(13)°			a = 3.3273 Å<br>b = 6.4281 Å<br>c = 3.6955 Å<br>β = 104.491°		
**Atomic positions**	Sc1	x	0.4779(2)	Sc1	x	0.4796
		y	0.25		y	0.2500
		z	0.13202(18)		z	0.1338
	N1	x	0.1061(6)	N1	x	0.1052
		y	0.5835(8)		y	0.5845
		z	0.4462(5)		z	0.4439
	N2	x	0.1897(7)	N2	x	0.1868
		y	0.5396(8)		y	0.5405
		z	0.1165(5)		z	0.1180
	N3	x	0.1740(10)	N3	x	0.1744
		y	0.25		y	0.2500
		z	0.5651(8)		z	0.5652

Supplementary figures

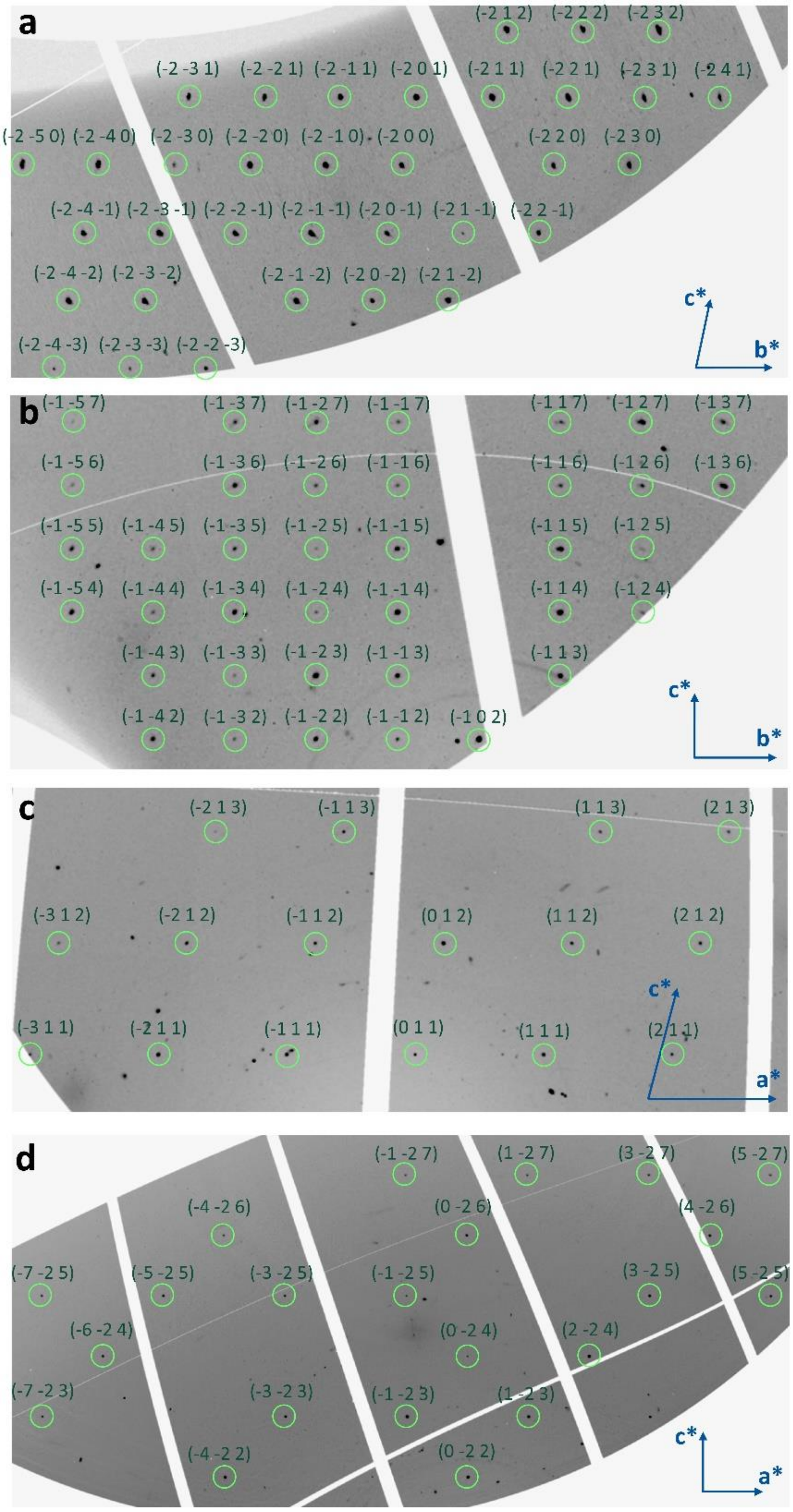

Supplementary Figure 1. The slices of the reciprocal space (also called unwarps) for a given (*hkl*) plane of a given crystallite, reconstructed from the experimental single-crystal XRD datasets in CrysAlis^{Pro} software, demonstrating the quality of single-crystal reflections: (a) (-2*kl*) plane of Sc_2N_6 at 78 GPa, (b) (-1*kl*) plane of Sc_2N_8 at 78 GPa, (c) (*h*1*l*) plane of ScN_5 at 96 GPa and (d) (*h*-2*l*) plane of Sc_4N_3 at 125 GPa. The green-encircled reflections correspond to the crystallites of (a) Sc_2N_6, (b) Sc_2N_8, (c) ScN_5, or (d) Sc_4N_3 whose structure was determined. The reflections that are not encircled belong to other crystallites of the same Sc-N phase or of other Sc-N phases present in multiphase multigrain samples.

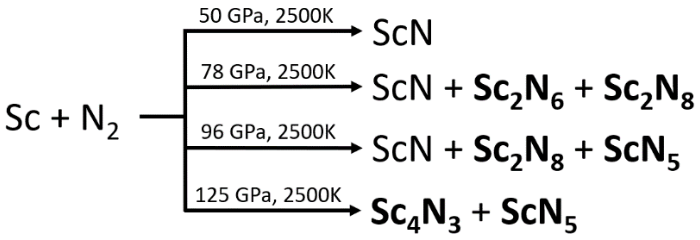

Supplementary Figure 2. Summary of the high-pressure high-temperature induced reactions of Sc and N_2 studied in this paper.

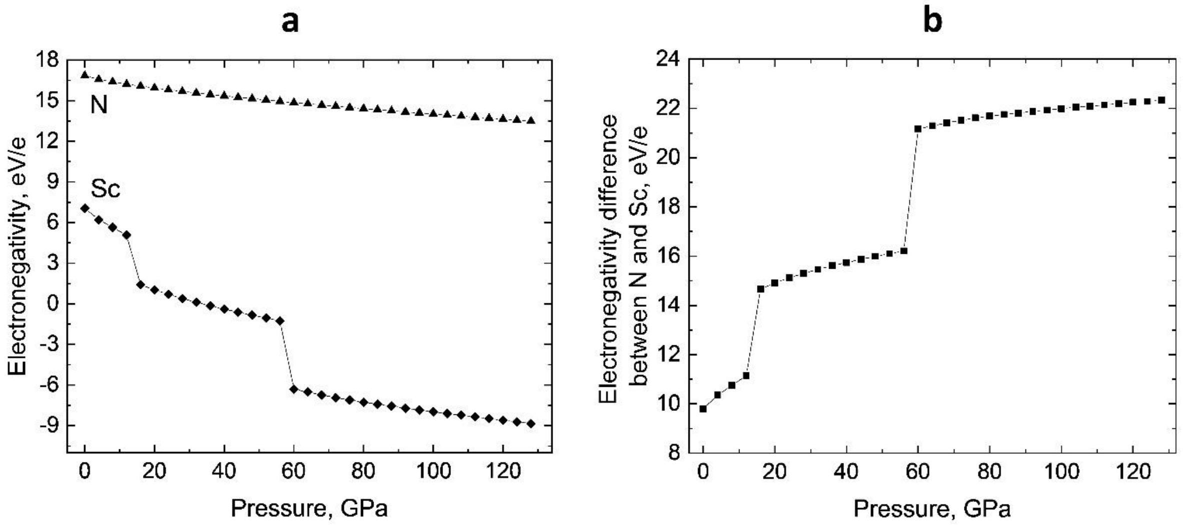

Supplementary Figure 3. Pressure dependence of the (a) electronegativity of N and Sc elements and (b) electronegativity difference between N and Sc according to [1], where the electronegativity is defined as the average electron energy. This scale might be roughly connected to the Pauling electronegativity scale, where 1 Pauling unit ≈ 6 eV/e.[1] At 60 GPa electronegativity difference between N and Sc increases by ~5 eV/e, changing from 16.2 to 21.2 eV/e.

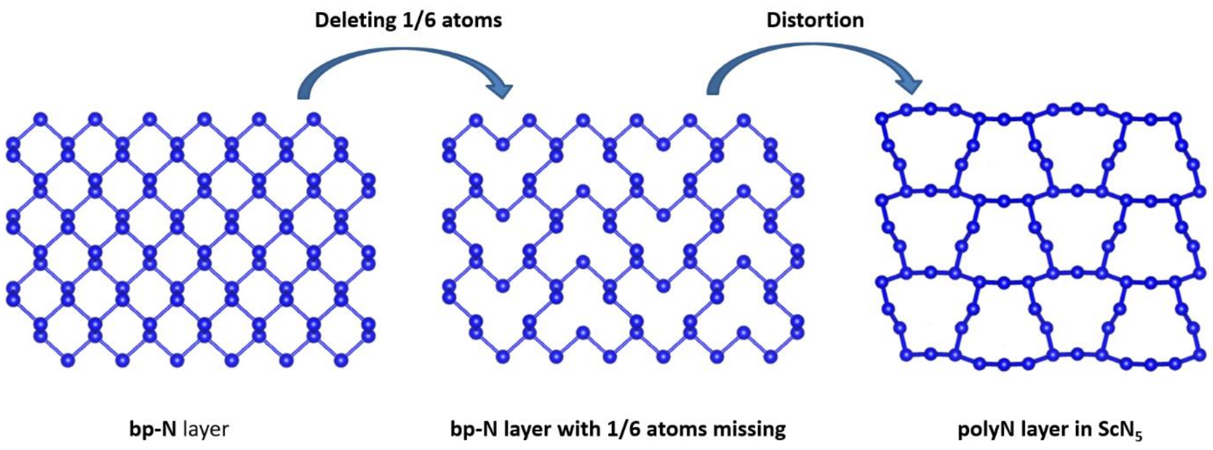

Supplementary Figure 4. The evolution of the bp-N layer to the polynitrogen layer in ScN_5.

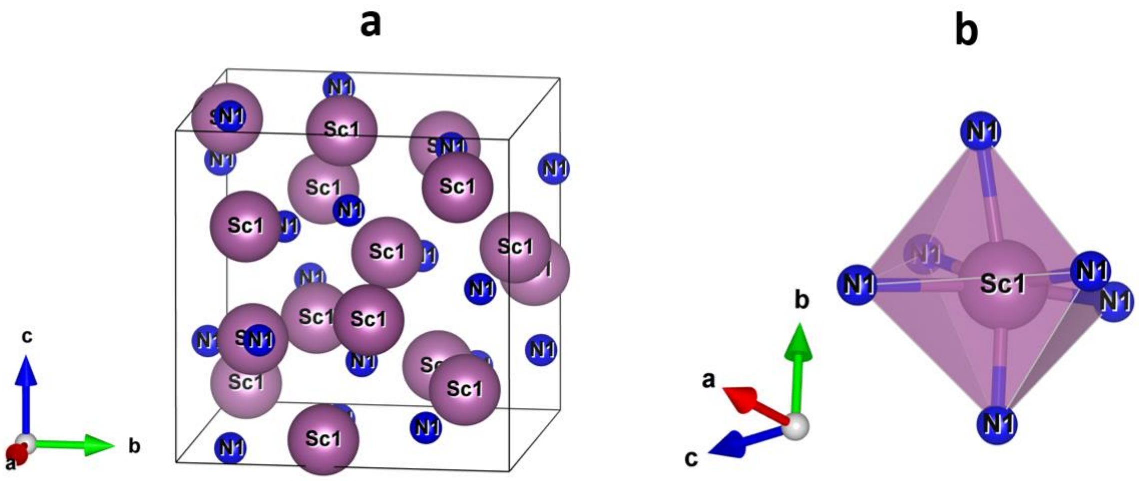

Supplementary Figure 5. Crystal structure of Sc_4N_3. Sc atoms are purple, N atoms are blue; grey thin lines outline the unit cell. (a) A general view of the crystal structure. (b) The coordination polyhedron of Sc atom: distorted octahedron with three Sc-N distances of 2.0332(7) Å and three Sc-N distances of 2.2396(7) Å at 125 GPa.

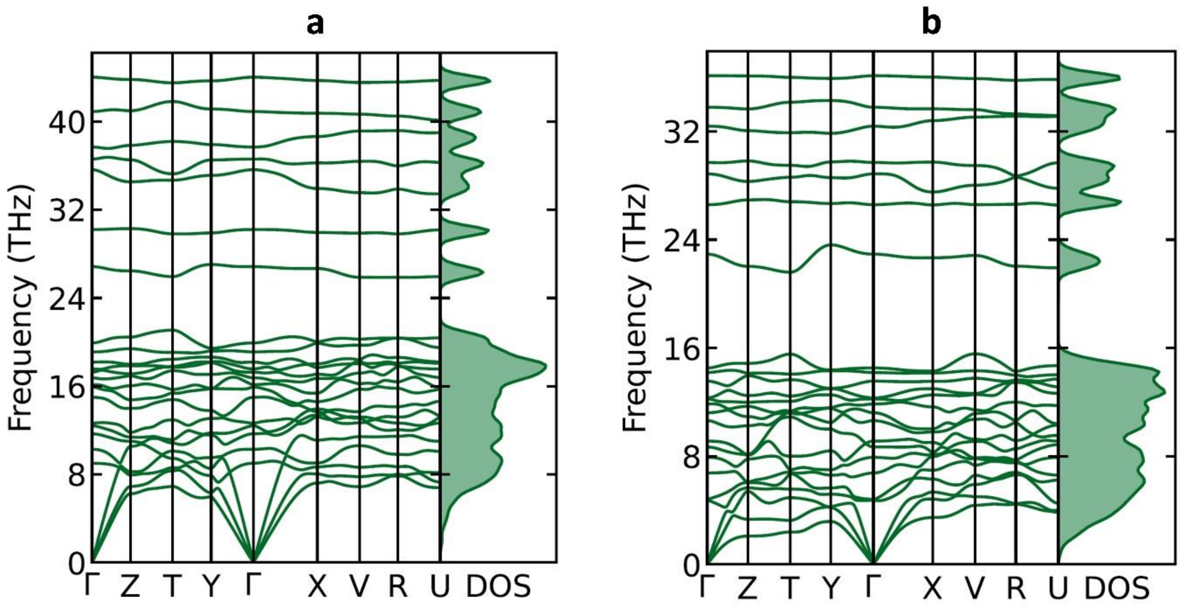

Supplementary Figure 6. Phonon dispersions of Sc_2N_6 at (a) 78 GPa and (b) 1 bar.

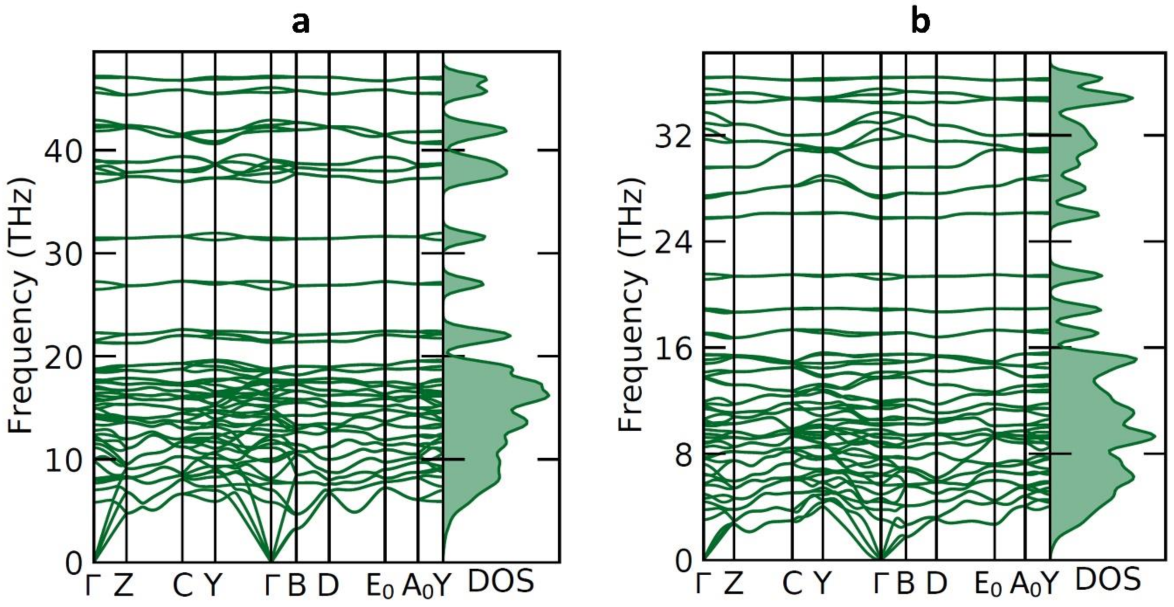

Supplementary Figure 7. Phonon dispersions of Sc_2N_8 at (a) 96 GPa and (b) 1 bar.

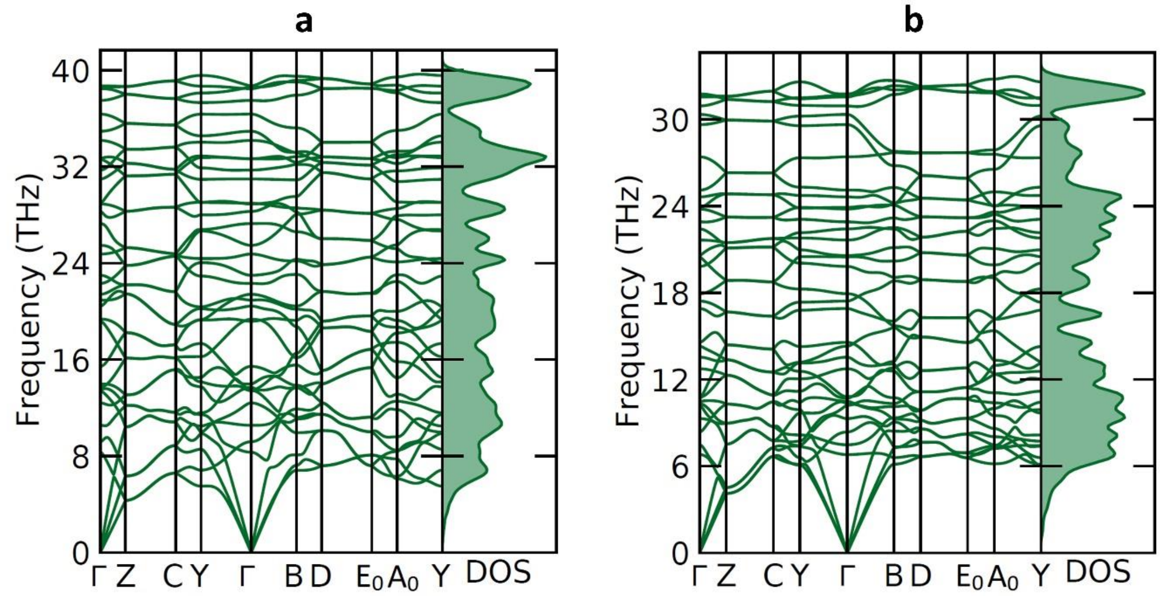

Supplementary Figure 8. Phonon dispersions of ScN_5 at (a) 96 GPa and (b) 1 bar.

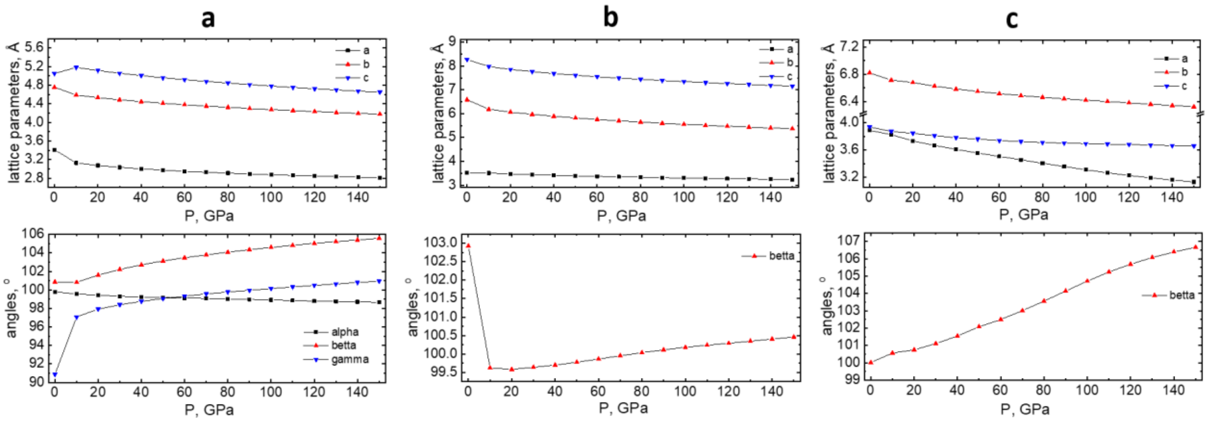

Supplementary Figure 9. The dependence of the unit cell parameters on pressure of (a) Sc_2N_6, (b) Sc_2N_8, and (c) ScN_5 obtained from DFT. The jump of lattice parameters of Sc_2N_6 and Sc_2N_8 between 1 bar and 10 GPa may indicate the isostructural phase transitions. A comparison of the corresponding DFT-relaxed crystal structures at 1 bar and 10 GPa shows that the geometry of N_6 and N_8 units does not change, while the scandium coordination environment changes significantly due to a significant increase of some Sc-N distances.

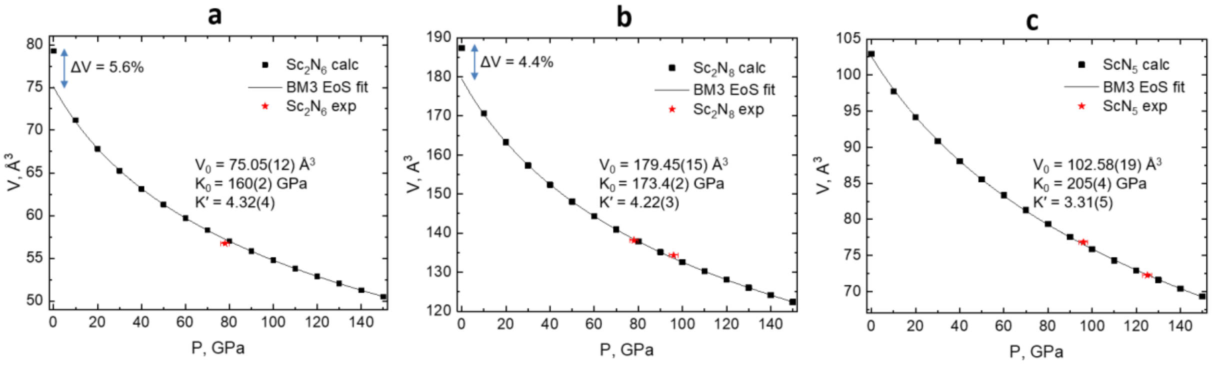

Supplementary Figure 10. Pressure dependence of the unit cell volume for (a) Sc_2N_6, (b) Sc_2N_8, and (c) ScN_5. The black square symbols represent calculated data points obtained from DFT, the red star symbols represent experimental data points obtained from SC-XRD data. The black lines are fits of the DFT data with the 3rd order Birch-Murnaghan equation of state. For Sc_2N_6 and Sc_2N_8 the point at 1 bar was not included in the fit.

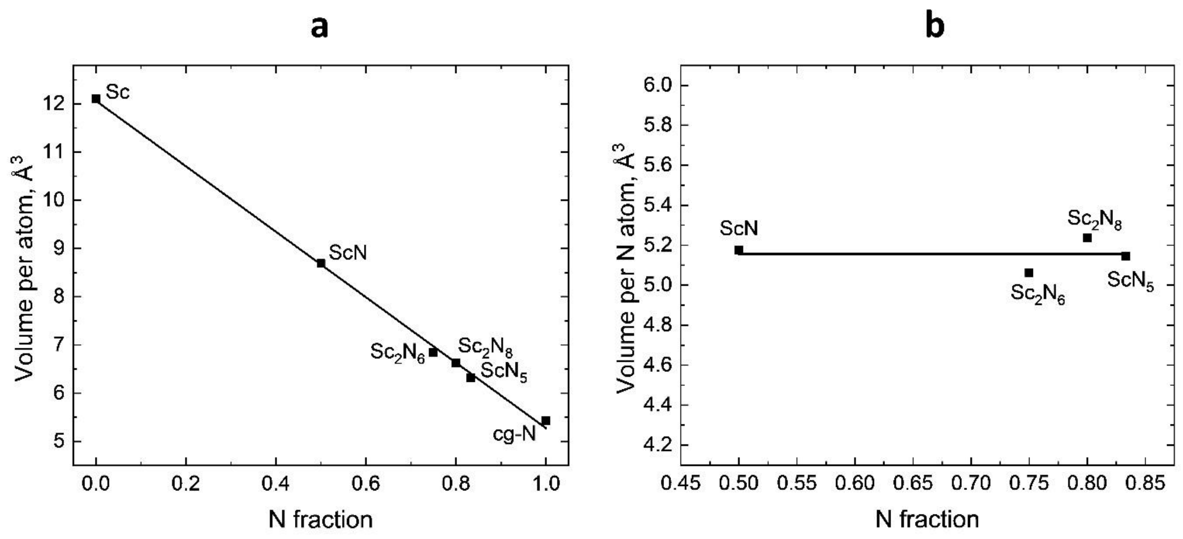

Supplementary Figure 11. (a) Volume per atom for scandium nitrides at 100 GPa. (b) Volume per nitrogen atom for scandium nitrides at 100 GPa. Note: the volume of a scandium atom was considered to be equal, in each phase, to the volume per atom in the scandium metal according to the published equation of state.[2]

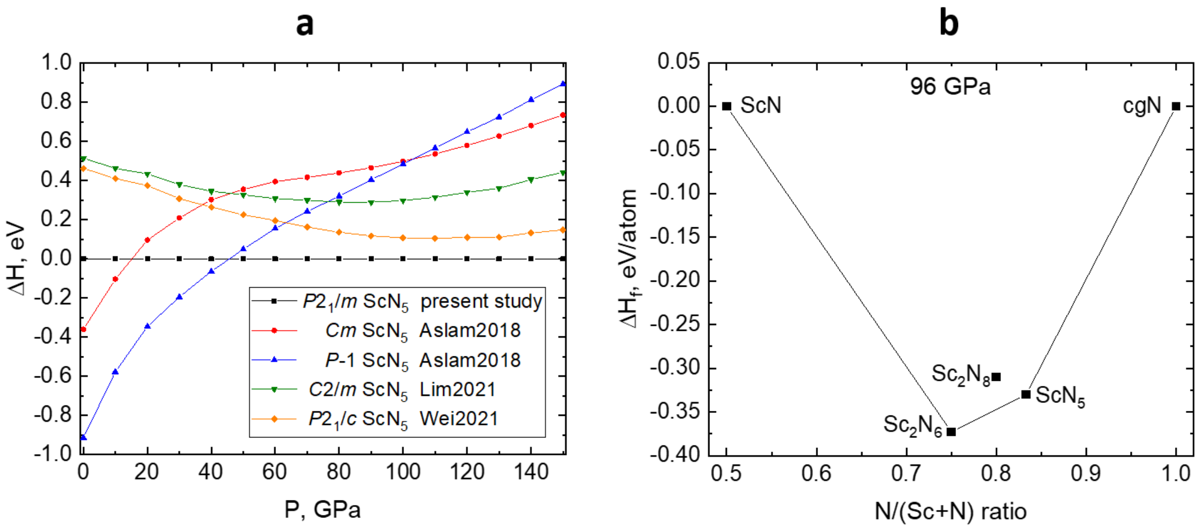

Supplementary Figure 12. (a) Calculated relative formation enthalpies of ScN_5 for various predicted structures (*Cm* ScN_5,[3] *P*-1 ScN_5,[3] *C*2/*m* ScN_5,[4] and *P*2$_1$/*c* ScN_5[5]) with respect to *P*2$_1$/*m* ScN_5 synthesized in the present study. (b) The calculated nitrogen-rich part of the convex hull in the Sc-N binary system for known scandium nitrides at 96 GPa. The phases Sc_2N_6 and ScN_5 lie on the convex hull and are thus thermodynamically stable, while Sc_2N_8 is metastable since it is out of the convex hull.

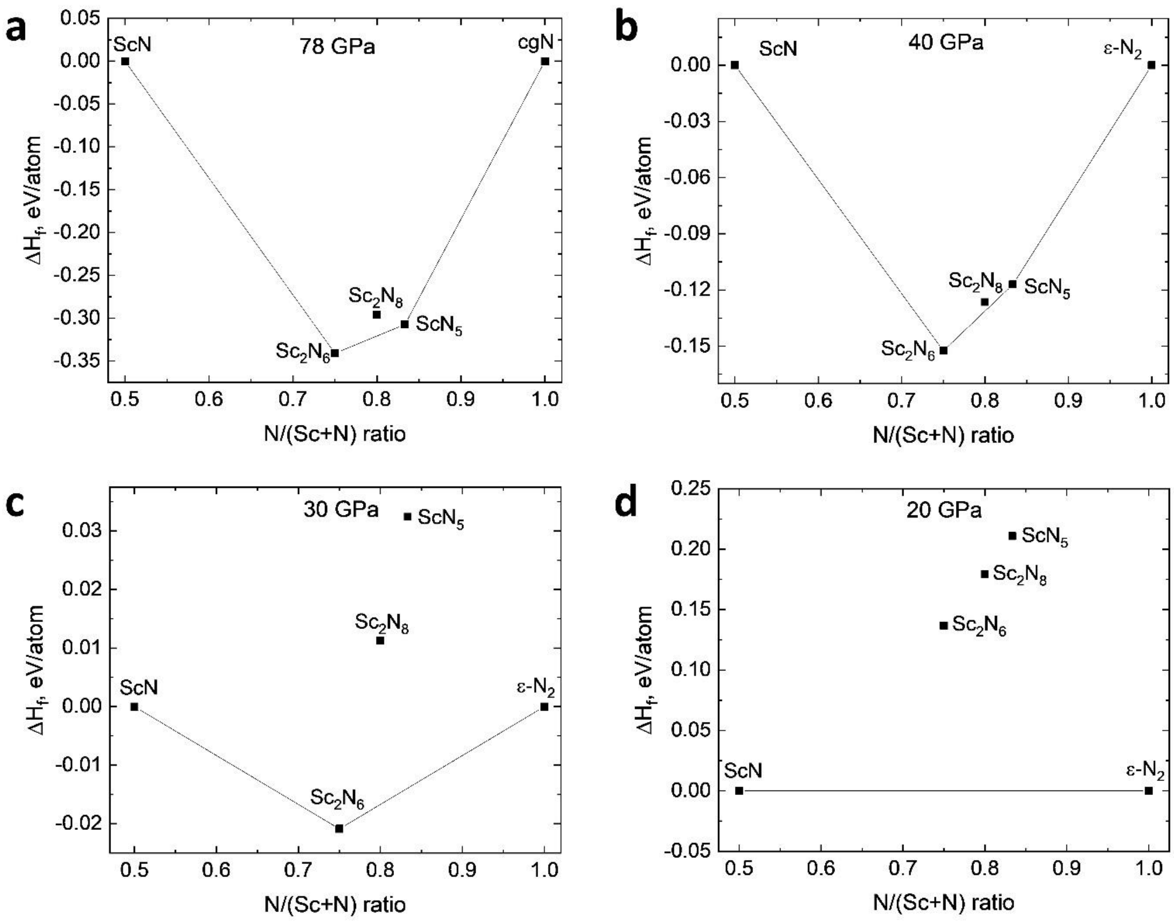

Supplementary Figure 13. The calculated nitrogen-rich part of the convex hull in the Sc-N binary system for known scandium nitrides at (a) 78 GPa, (b) 40 GPa, (c) 30 GPa and (d) 20 GPa

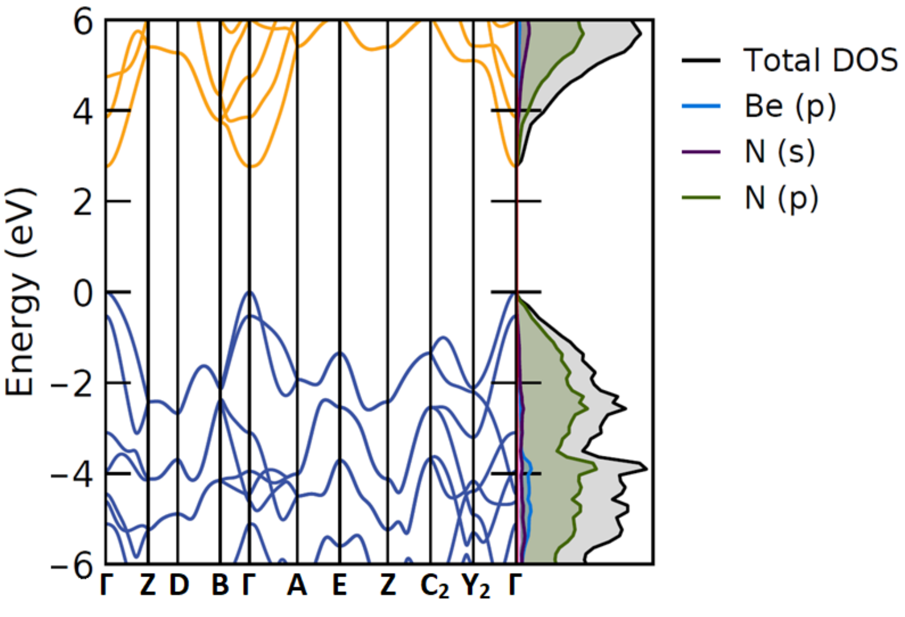

Supplementary Figure 14. The electron density of states of m-BeN_4 [6]

Supplementary References

1. Rahm, M., Cammi, R., Ashcroft, N. W. & Hoffmann, R. Squeezing All Elements in the Periodic Table: Electron Configuration and Electronegativity of the Atoms under Compression. *J. Am. Chem. Soc.* **141**, 10253–10271 (2019).

2. Fujihisa, H. *et al.* Incommensurate composite crystal structure of scandium-II. *Phys. Rev. B* **72**, 132103 (2005).

3. Aslam, M. A. & Ding, Z. J. Prediction of Thermodynamically Stable Compounds of the Sc-N System under High Pressure. *ACS Omega* **3**, 11477–11485 (2018).

4. Lin, J. *et al.* Stable nitrogen-rich scandium nitrides and their bonding features under ambient conditions. *Phys. Chem. Chem. Phys.* **23**, 6863–6870 (2021).

5. Wei, S. *et al.* A novel high-pressure phase of ScN_5 with higher stability predicted from first-principles calculations. *J. Phys. Condens. Matter* **33**, 475401 (2021).

6. Bykov, M. *et al.* High-Pressure Synthesis of Dirac Materials: Layered van der Waals Bonded BeN_4 Polymorph. *Phys. Rev. Lett.* **126**, 175501 (2021).
