# Supplementary material for: Stabilization of N6 and N8 anionic units and 2D polynitrogen layers in high-pressure scandium polynitrides
Source: Nat Commun. 2024 Mar 12;15:2244. doi: 10.1038/s41467-024-46313-9 (PMC11636835; doi:10.1038/s41467-024-46313-9)

checkCIF/PLATON report

Structure factors have been supplied for datablock(s) Sc4N3_125GPa

THIS REPORT IS FOR GUIDANCE ONLY. IF USED AS PART OF A REVIEW PROCEDURE FOR PUBLICATION, IT SHOULD NOT REPLACE THE EXPERTISE OF AN EXPERIENCED CRYSTALLOGRAPHIC REFEREE.

No syntax errors found. CIF dictionary Interpreting this report

Datablock: Sc4N3_125GPa

Bond precision: Sc-Sc = 0.0009 A Wavelength=0.41000

Cell:	a=6.1613(13)	b=6.1613(13)	c=6.1613(13)
	alpha=90	beta=90	gamma=90
Temperature:	293 K		

	Calculated	Reported
Volume	233.89(15)	233.89(15)
Space group	I -4 3 d	I -4 3 d
Hall group	I -4bd 2c 3	I -4bd 2c 3
Moiety formula	N3 Sc4	N3 Sc4
Sum formula	N3 Sc4	N3 Sc4
Mr	221.87	221.87
Dx,g cm-3	6.301	6.301
Z	4	4
Mu (mm-1)	2.213	2.232
F000	420.0	420.0
F000’	421.66	
h,k,lmax	10,10,10	9,6,9
Nref	109[65]	83
Tmin,Tmax	0.998,0.998	0.647,1.000
Tmin’	0.998	

Correction method= # Reported T Limits: Tmin=0.647 Tmax=1.000
AbsCorr = MULTI-SCAN

Data completeness= 1.28/0.76 Theta(max)= 20.999

R(reflections)= 0.0266(80) wR2(reflections)= 0.0638(83)

S = 1.078 Npar= 6

The following ALERTS were generated. Each ALERT has the format
test-name_ALERT_alert-type_alert-level.
Click on the hyperlinks for more details of the test.

Alert level C

```
PLAT034_ALERT_1_C No Flack Parameter Given. Z > Si, NonCentro ....     Please Do !
PLAT975_ALERT_2_C Check Calcd Resid. Dens.  0.41Ang From N1      .       0.54 eA-3
```

Alert level G

```
ABSMU01_ALERT_1_G  Calculation of _exptl_absorpt_correction_mu
                has not performed for this radiation type.
PLAT004_ALERT_5_G Polymeric Structure Found with Maximum Dimension          3 Info
PLAT012_ALERT_1_G N.O.K.   _shelx_res_checksum Found in CIF ......     Please Check
PLAT013_ALERT_1_G N.O.K.   _shelx_hkl_checksum Found in CIF ......     Please Check
PLAT092_ALERT_4_G Check: Wavelength Given is not Cu,Ga,Mo,Ag,In Ka    0.41000 Ang.
PLAT199_ALERT_1_G Reported _cell_measurement_temperature ..... (K)        293 Check
PLAT200_ALERT_1_G Reported   _diffrn_ambient_temperature ..... (K)        293 Check
PLAT912_ALERT_4_G Missing # of FCF Reflections Above STh/L=  0.600          8 Note
PLAT915_ALERT_3_G No Flack x Check Done: Low Friedel Pair Coverage         64 %
PLAT933_ALERT_2_G Number of HKL-OMIT Records in Embedded .res File          1 Note
PLAT941_ALERT_3_G Average HKL Measurement Multiplicity ..........         4.5 Low
PLAT951_ALERT_5_G Calculated (ThMax) and CIF-Reported Kmax Differ           4 Units
PLAT984_ALERT_1_G The  N-f'=   -0.0016 Deviates from the B&C-Value     0.0004 Check
PLAT984_ALERT_1_G The Sc-f'=    0.0809 Deviates from the B&C-Value     0.1087 Check
```

```
   0 ALERT level A = Most likely a serious problem - resolve or explain
   0 ALERT level B = A potentially serious problem, consider carefully
   2 ALERT level C = Check. Ensure it is not caused by an omission or oversight
  14 ALERT level G = General information/check it is not something unexpected

   8 ALERT type 1 CIF construction/syntax error, inconsistent or missing data
   2 ALERT type 2 Indicator that the structure model may be wrong or deficient
   2 ALERT type 3 Indicator that the structure quality may be low
   2 ALERT type 4 Improvement, methodology, query or suggestion
   2 ALERT type 5 Informative message, check
```

It is advisable to attempt to resolve as many as possible of the alerts in all categories. Often the minor alerts point to easily fixed oversights, errors and omissions in your CIF or refinement strategy, so attention to these fine details can be worthwhile. In order to resolve some of the more serious problems it may be necessary to carry out additional measurements or structure refinements. However, the purpose of your study may justify the reported deviations and the more serious of these should normally be commented upon in the discussion or experimental section of a paper or in the "special_details" fields of the CIF. checkCIF was carefully designed to identify outliers and unusual parameters, but every test has its limitations and alerts that are not important in a particular case may appear. Conversely, the absence of alerts does not guarantee there are no aspects of the results needing attention. It is up to the individual to critically assess their own results and, if necessary, seek expert advice.

Publication of your CIF in IUCr journals

A basic structural check has been run on your CIF. These basic checks will be run on all CIFs submitted for publication in IUCr journals (*Acta Crystallographica*, *Journal of Applied Crystallography*, *Journal of Synchrotron Radiation*); however, if you intend to submit to *Acta Crystallographica Section C* or *E* or *IUCrData*, you should make sure that full publication checks are run on the final version of your CIF prior to submission.

Publication of your CIF in other journals

Please refer to the *Notes for Authors* of the relevant journal for any special instructions relating to CIF submission.

PLATON version of 28/11/2022; check.def file version of 28/11/2022

Datablock Sc4N3_125GPa - ellipsoid plot

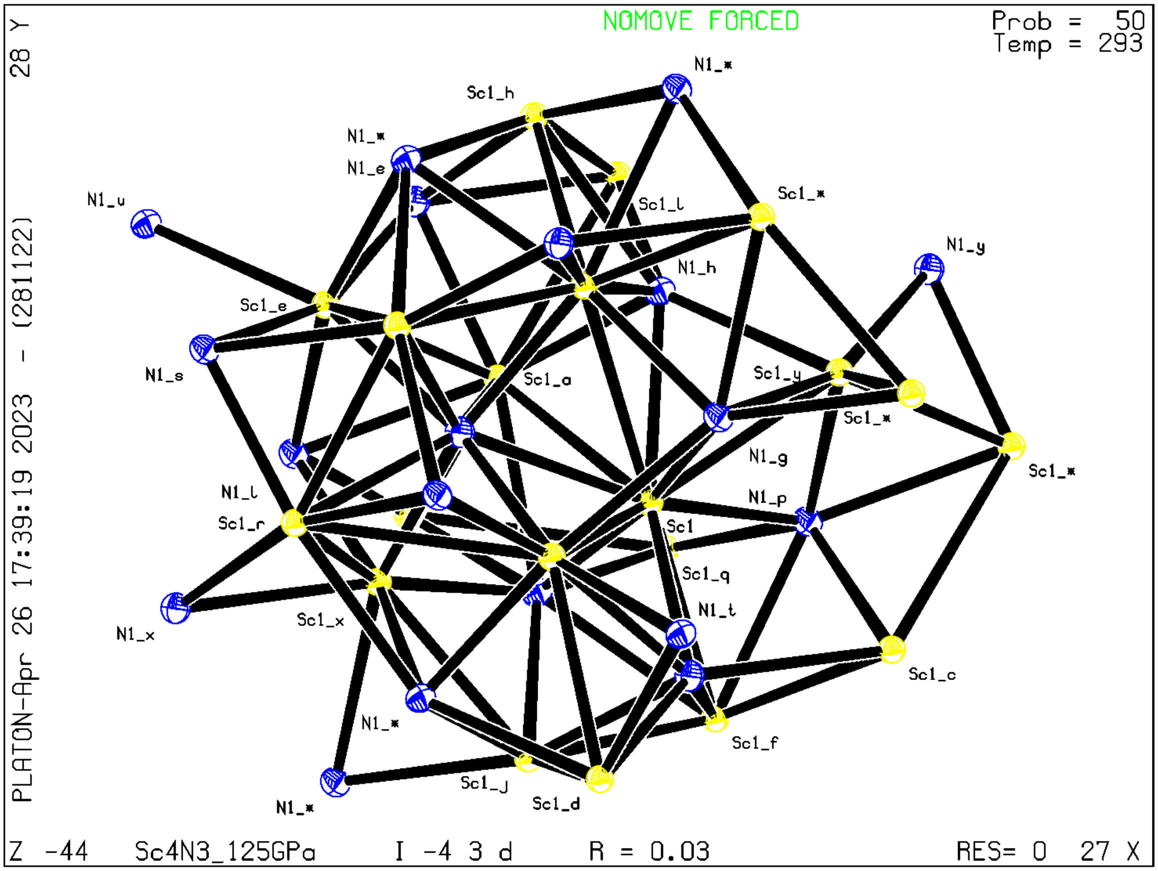

Supplement: Supplementary file 3 — Source Data [file 41467_2024_46313_MOESM3_ESM.zip › Sc4N3_125GPa_checkcif.pdf]
