# Supplementary material for: Stabilization of N6 and N8 anionic units and 2D polynitrogen layers in high-pressure scandium polynitrides
Source: Nat Commun. 2024 Mar 12;15:2244. doi: 10.1038/s41467-024-46313-9 (PMC11636835; doi:10.1038/s41467-024-46313-9)

# checkCIF/PLATON report

Structure factors have been supplied for datablock(s) ScN_50GPa

THIS REPORT IS FOR GUIDANCE ONLY. IF USED AS PART OF A REVIEW PROCEDURE FOR PUBLICATION, IT SHOULD NOT REPLACE THE EXPERTISE OF AN EXPERIENCED CRYSTALLOGRAPHIC REFEREE.

No syntax errors found.    CIF dictionary    Interpreting this report

# Datablock: ScN_50GPa

---

Bond precision: Sc- N = 0.0003 A    Wavelength=0.29521

| | | | |
|---|---|---|---|
| Cell: | a=4.2492(7) | b=4.2492(7) | c=4.2492(7) |
| | alpha=90 | beta=90 | gamma=90 |
| Temperature: | 293 K | | |

| | Calculated | Reported |
|---|---|---|
| Volume | 76.72(4) | 76.72(4) |
| Space group | F m -3 m | F m -3 m |
| Hall group | -F 4 2 3 | -F 4 2 3 |
| Moiety formula | N Sc | N Sc |
| Sum formula | N Sc | N Sc |
| Mr | 58.97 | 58.97 |
| Dx,g cm-3 | 5.106 | 5.105 |
| Z | 4 | 4 |
| Mu (mm-1) | 0.692 | 0.709 |
| F000 | 112.0 | 112.0 |
| F000’ | 112.18 | |
| h,k,lmax | 7,7,7 | 6,6,4 |
| Nref | 22 | 19 |
| Tmin,Tmax | 0.996,0.996 | |
| Tmin’ | 0.996 | |

Correction method= Not given

Data completeness= 0.864    Theta(max)= 14.507

R(reflections)= 0.0341( 19)    wR2(reflections)= 0.0772( 19)

S = 1.202    Npar= 3

The following ALERTS were generated. Each ALERT has the format
**test-name_ALERT_alert-type_alert-level.**
Click on the hyperlinks for more details of the test.

### Alert level B

```
PLAT088_ALERT_3_B Poor Data / Parameter Ratio ...................       6.33 Note
```

**Author Response: This measurement was performed at high pressure. The diamond anvil cell metallic body typically shadows more than 60% of the reflections.**

```
PLAT113_ALERT_2_B ADDSYM Suggests Possible Pseudo/New  Space Group      Pm-3m Check
              Check Model Parameter Symmmetry for Reflection Data Support
```

**Author Response: Fm-3m spacegroup is correct.**

### Alert level G

```
ABSMU01_ALERT_1_G  Calculation of _exptl_absorpt_correction_mu
                not performed for this radiation type.
PLAT004_ALERT_5_G Polymeric Structure Found with Maximum Dimension          2 Info
PLAT012_ALERT_1_G N.O.K.   _shelx_res_checksum Found in CIF ......     Please Check
PLAT092_ALERT_4_G Check: Wavelength Given is not Cu,Ga,Mo,Ag,In Ka    0.29521 Ang.
PLAT110_ALERT_2_G ADDSYM Detects Potential Lattice Translation ...          ? Check
PLAT112_ALERT_2_G ADDSYM Detects New (Pseudo) Symm. Elem         I        100 %Fit
PLAT116_ALERT_2_G ADDSYM Included (Pseudo) Lattice Translation ...     Please Check
PLAT199_ALERT_1_G Reported _cell_measurement_temperature ..... (K)        293 Check
PLAT200_ALERT_1_G Reported   _diffrn_ambient_temperature ..... (K)        293 Check
PLAT912_ALERT_4_G Missing # of FCF Reflections Above STh/L=  0.600          2 Note
PLAT941_ALERT_3_G Average HKL Measurement Multiplicity ..........         2.1 Low
PLAT952_ALERT_5_G Calculated (ThMax) and CIF-Reported Lmax Differ.          3 Units
PLAT955_ALERT_1_G Reported (CIF) and Actual (FCF) Lmax Differ by .          2 Units
PLAT961_ALERT_5_G Dataset Contains no Negative Intensities .......     Please Check
PLAT984_ALERT_1_G The  N-f'=   -0.0031 Deviates from the B&C-Value    -0.0011 Check
PLAT984_ALERT_1_G The Sc-f'=    0.0242 Deviates from the B&C-Value     0.0525 Check
```

---

0 **ALERT level A** = Most likely a serious problem - resolve or explain
2 **ALERT level B** = A potentially serious problem, consider carefully
0 **ALERT level C** = Check. Ensure it is not caused by an omission or oversight
16 **ALERT level G** = General information/check it is not something unexpected

7 ALERT type 1 CIF construction/syntax error, inconsistent or missing data
4 ALERT type 2 Indicator that the structure model may be wrong or deficient
2 ALERT type 3 Indicator that the structure quality may be low
2 ALERT type 4 Improvement, methodology, query or suggestion
3 ALERT type 5 Informative message, check

It is advisable to attempt to resolve as many as possible of the alerts in all categories. Often the minor alerts point to easily fixed oversights, errors and omissions in your CIF or refinement strategy, so attention to these fine details can be worthwhile. In order to resolve some of the more serious problems it may be necessary to carry out additional measurements or structure refinements. However, the purpose of your study may justify the reported deviations and the more serious of these should normally be commented upon in the discussion or experimental section of a paper or in the "special_details" fields of the CIF. checkCIF was carefully designed to identify outliers and unusual parameters, but every test has its limitations and alerts that are not important in a particular case may appear. Conversely, the absence of alerts does not guarantee there are no aspects of the results needing attention. It is up to the individual to critically assess their own results and, if necessary, seek expert advice.

**Publication of your CIF in IUCr journals**

A basic structural check has been run on your CIF. These basic checks will be run on all CIFs submitted for publication in IUCr journals (*Acta Crystallographica*, *Journal of Applied Crystallography*, *Journal of Synchrotron Radiation*); however, if you intend to submit to *Acta Crystallographica Section C* or *E* or *IUCrData*, you should make sure that full publication checks are run on the final version of your CIF prior to submission.

**Publication of your CIF in other journals**

Please refer to the *Notes for Authors* of the relevant journal for any special instructions relating to CIF submission.

---

**PLATON version of 28/11/2022; check.def file version of 28/11/2022**

**Datablock ScN_50GPa** - ellipsoid plot

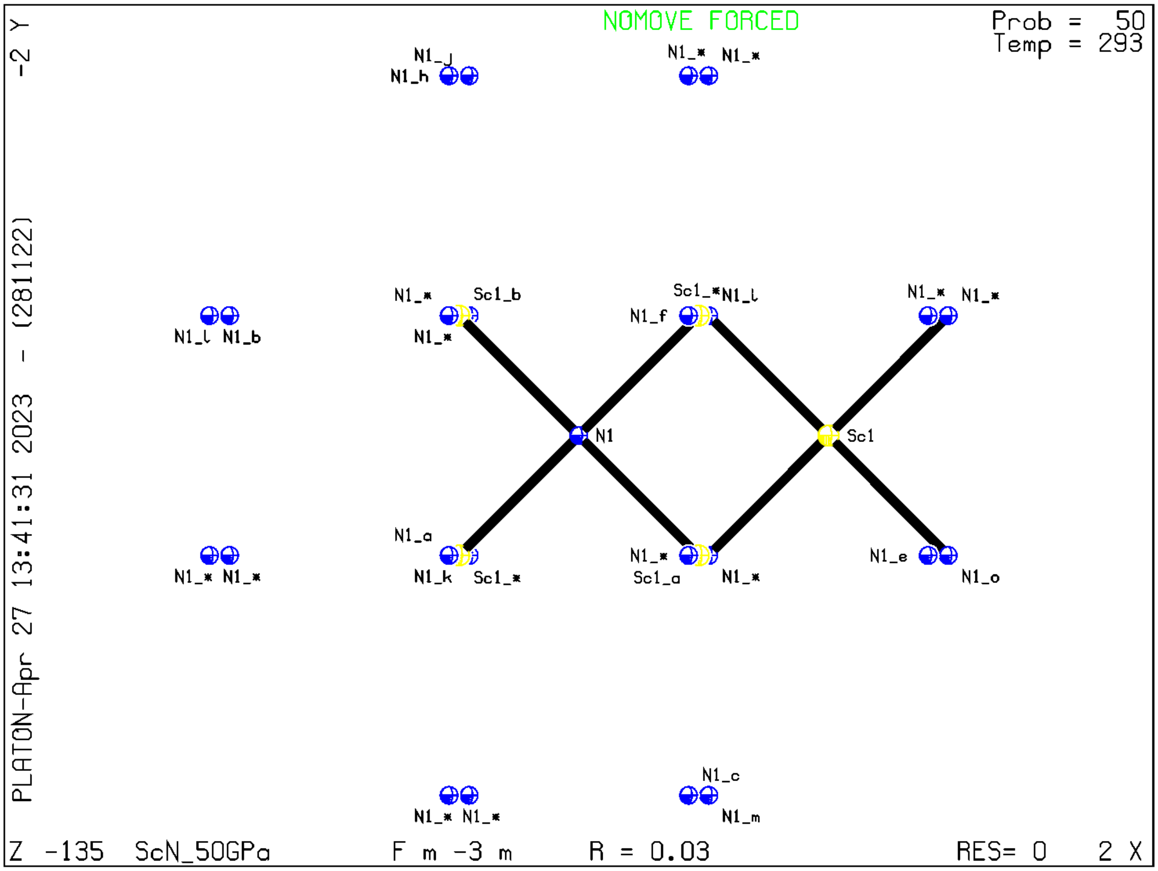

Supplement: Supplementary file 3 — Source Data [file ScN_50GPa_checkcif.pdf › ScN_50GPa_checkcif.pdf]
