# Supplementary material for: Stabilization of N6 and N8 anionic units and 2D polynitrogen layers in high-pressure scandium polynitrides
Source: Nat Commun. 2024 Mar 12;15:2244. doi: 10.1038/s41467-024-46313-9 (PMC11636835; doi:10.1038/s41467-024-46313-9)

checkCIF/PLATON report

Structure factors have been supplied for datablock(s) ScN5_96GPa

THIS REPORT IS FOR GUIDANCE ONLY. IF USED AS PART OF A REVIEW PROCEDURE FOR PUBLICATION, IT SHOULD NOT REPLACE THE EXPERTISE OF AN EXPERIENCED CRYSTALLOGRAPHIC REFEREE.

No syntax errors found. CIF dictionary Interpreting this report

Datablock: ScN5_96GPa

Bond precision:	N- N = 0.0060 A	Wavelength=0.28430	
Cell:	a=3.3225(6)	b=6.440(3)	c=3.7067(4)
	alpha=90	beta=104.339(13)	gamma=90
Temperature:	293 K		

	Calculated	Reported
Volume	76.84(4)	76.84(4)
Space group	P 21/m	P 1 21/m 1
Hall group	-P 2yb	-P 2yb
Moiety formula	N5 Sc	N5 Sc
Sum formula	N5 Sc	N5 Sc
Mr	115.01	115.01
Dx,g cm-3	4.971	4.971
Z	2	2
Mu (mm-1)	0.366	0.373
F000	112.0	112.0
F000'	112.03	
h,k,lmax	7,13,7	5,7,6
Nref	821	264
Tmin,Tmax	1.000,1.000	0.453,1.000
Tmin'	1.000	

Correction method= # Reported T Limits: Tmin=0.453 Tmax=1.000
AbsCorr = MULTI-SCAN

Data completeness= 0.322 Theta(max)= 17.664

R(reflections)= 0.0363(202) wR2(reflections)= 0.0877(264)

S = 1.008 Npar= 31

The following ALERTS were generated. Each ALERT has the format
test-name_ALERT_alert-type_alert-level.
Click on the hyperlinks for more details of the test.

Alert level A

PLAT029_ALERT_3_A _diffrn_measured_fraction_theta_full value Low . 0.547 Why?

Author Response: This measurement was performed at high pressure which, due to the high pressure apparatus, limits the theta range. Indeed, the diamond anvil cell metallic body typically shadows more than 60% of the reflections.

Alert level B

PLAT911_ALERT_3_B Missing FCF Refl Between Thmin & STh/L= 0.600 68 Report

Author Response: This measurement was performed at high pressure which, due to the high pressure apparatus, limits the theta range. Indeed, the diamond anvil cell metallic body typically shadows more than 60% of the reflections.

Alert level C

PLAT088_ALERT_3_C Poor Data / Parameter Ratio .... 8.52 Note

Alert level G

```
ABSMU01_ALERT_1_G  Calculation of _exptl_absorpt_correction_mu
                not performed for this radiation type.
PLAT004_ALERT_5_G Polymeric Structure Found with Maximum Dimension         3 Info
PLAT012_ALERT_1_G N.O.K.   _shelx_res_checksum Found in CIF ......    Please Check
PLAT013_ALERT_1_G N.O.K.   _shelx_hkl_checksum Found in CIF ......    Please Check
PLAT092_ALERT_4_G Check: Wavelength Given is not Cu,Ga,Mo,Ag,In Ka   0.28430 Ang.
PLAT199_ALERT_1_G Reported _cell_measurement_temperature ..... (K)       293 Check
PLAT200_ALERT_1_G Reported   _diffrn_ambient_temperature ..... (K)       293 Check
PLAT912_ALERT_4_G Missing # of FCF Reflections Above STh/L=  0.600       192 Note
PLAT933_ALERT_2_G Number of HKL-OMIT Records in Embedded .res File         5 Note
PLAT941_ALERT_3_G Average HKL Measurement Multiplicity ...........       1.7 Low
PLAT950_ALERT_5_G Calculated (ThMax) and CIF-Reported Hmax Differ          2 Units
PLAT951_ALERT_5_G Calculated (ThMax) and CIF-Reported Kmax Differ          6 Units
PLAT956_ALERT_1_G Calculated (ThMax) and Actual (FCF) Hmax Differ          2 Units
PLAT957_ALERT_1_G Calculated (ThMax) and Actual (FCF) Kmax Differ          6 Units
PLAT984_ALERT_1_G The  N-f'=   -0.0033 Deviates from the B&C-Value   -0.0013 Check
PLAT984_ALERT_1_G The Sc-f'=    0.0191 Deviates from the B&C-Value    0.0474 Check
```

1 **ALERT level A** = Most likely a serious problem - resolve or explain
1 **ALERT level B** = A potentially serious problem, consider carefully
1 **ALERT level C** = Check. Ensure it is not caused by an omission or oversight
16 **ALERT level G** = General information/check it is not something unexpected

```
9 ALERT type 1 CIF construction/syntax error, inconsistent or missing data
1 ALERT type 2 Indicator that the structure model may be wrong or deficient
4 ALERT type 3 Indicator that the structure quality may be low
2 ALERT type 4 Improvement, methodology, query or suggestion
3 ALERT type 5 Informative message, check
```

It is advisable to attempt to resolve as many as possible of the alerts in all categories. Often the minor alerts point to easily fixed oversights, errors and omissions in your CIF or refinement strategy, so attention to these fine details can be worthwhile. In order to resolve some of the more serious problems it may be necessary to carry out additional measurements or structure refinements. However, the purpose of your study may justify the reported deviations and the more serious of these should normally be commented upon in the discussion or experimental section of a paper or in the "special_details" fields of the CIF. checkCIF was carefully designed to identify outliers and unusual parameters, but every test has its limitations and alerts that are not important in a particular case may appear. Conversely, the absence of alerts does not guarantee there are no aspects of the results needing attention. It is up to the individual to critically assess their own results and, if necessary, seek expert advice.

Publication of your CIF in IUCr journals

A basic structural check has been run on your CIF. These basic checks will be run on all CIFs submitted for publication in IUCr journals (*Acta Crystallographica*, *Journal of Applied Crystallography*, *Journal of Synchrotron Radiation*); however, if you intend to submit to *Acta Crystallographica Section C* or *E* or *IUCrData*, you should make sure that full publication checks are run on the final version of your CIF prior to submission.

Publication of your CIF in other journals

Please refer to the *Notes for Authors* of the relevant journal for any special instructions relating to CIF submission.

PLATON version of 28/11/2022; check.def file version of 28/11/2022

Datablock ScN5_96GPa - ellipsoid plot

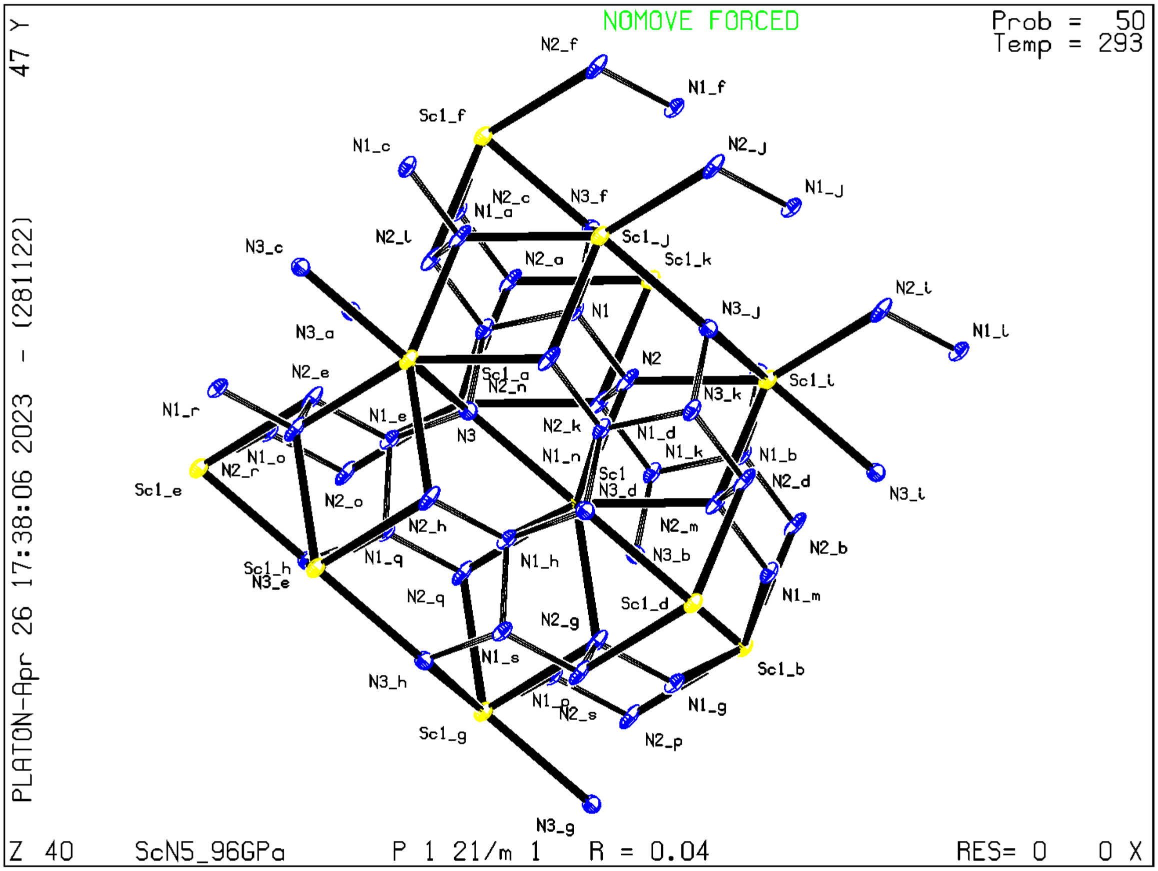

Supplement: Supplementary file 3 — Source Data [file ScN5_96GPa_checkcif.pdf › ScN5_96GPa_checkcif.pdf]
